# Two Hydroxyproline Galactosyltransferases, GALT5 and GALT2, Function in Arabinogalactan-Protein Glycosylation, Growth and Development in Arabidopsis

**DOI:** 10.1371/journal.pone.0125624

**Published:** 2015-05-14

**Authors:** Debarati Basu, Wuda Wang, Siyi Ma, Taylor DeBrosse, Emily Poirier, Kirk Emch, Eric Soukup, Lu Tian, Allan M. Showalter

**Affiliations:** Molecular and Cellular Biology Program, Department of Environmental and Plant Biology, Ohio University, Athens, Ohio, United States of America; Iowa State University, UNITED STATES

## Abstract

Hydroxyproline-O-galactosyltransferase (GALT) initiates O-glycosylation of arabinogalactan-proteins (AGPs). We previously characterized GALT2 (At4g21060), and now report on functional characterization of GALT5 (At1g74800). GALT5 was identified using heterologous expression in *Pichia* and an *in vitro* GALT assay. Product characterization showed GALT5 specifically adds galactose to hydroxyproline in AGP protein backbones. Functions of GALT2 and GALT5 were elucidated by phenotypic analysis of single and double mutant plants. Allelic *galt5* and *galt2* mutants, and particularly *galt2 galt5* double mutants, demonstrated lower GALT activities and reductions in β-Yariv-precipitated AGPs compared to wild type. Mutant plants showed pleiotropic growth and development phenotypes (defects in root hair growth, root elongation, pollen tube growth, flowering time, leaf development, silique length, and inflorescence growth), which were most severe in the double mutants. Conditional mutant phenotypes were also observed, including salt-hypersensitive root growth and root tip swelling as well as reduced inhibition of pollen tube growth and root growth in response to β-Yariv reagent. These mutants also phenocopy mutants for an AGP, SOS5, and two cell wall receptor-like kinases, FEI1 and FEI2, which exist in a genetic signaling pathway. In summary, GALT5 and GALT2 function as redundant GALTs that control AGP *O*-glycosylation, which is essential for normal growth and development.

## Introduction

The fundamental processes that underpin plant growth and development depend crucially on the action and assembly of gene products designed to form the cell wall [1]. Cell walls are composed of cellulose, hemicellulose, and pectin, along with protein and lignin [2]. Wall proteins have emerged as essential components because of their contribution to wall architecture and function [3]. Among the cell wall proteins, the hydroxyproline-rich glycoprotein (HRGP) superfamily constitutes the most abundant and diverse group of cell wall glycoproteins [4]. The HRGP superfamily is composed of a spectrum of molecules, ranging from lightly glycosylated proline-rich proteins to highly glycosylated arabinogalactan-proteins (AGPs) with the moderately glycosylated extensins in between these two extremes [5].

AGPs are ubiquitous in the plant kingdom and are expressed in virtually all cells, either at the cell surface as part of the plasma membrane, the cell wall, or as extracellular secretions [6–9]. AGPs are distinguished by their abundance of hydroxyproline (Hyp), alanine, serine and threonine residues in the protein backbone, the occurrence of Ala-Hyp, Ser-Hyp, and/or Thr-Hyp dipeptide repeats, the presence of type II arabinogalactan (AG) polysaccharide side chains covalently attached to the Hyp residues, and their ability to interact with a reddish-brown chemical dye called β-Yariv reagent. The polysaccharide side chains attached to peptidyl Hyp are composed of β-1,3-galactan backbones decorated with β-1,6-galactose side chains that are further decorated with α-arabinose as well as other sugars, such as β-(methyl)glucuronic acid, α-rhamnose, and α-fucose, which are present in lesser amounts [10], [11]. An alternate model of AGP polysaccharide structure supported by nmr data indicates the galactan backbone consists of repeating trigalactosyl blocks, containing two β-1,3-galactoses attached to β-1,6-galactose, with these blocks being decorated with side chains containing β-galactose and α-arabinose along with other sugars [12–13]. One or more glycosyltransferases are thought to be responsible for adding each sugar in the AG polysaccharide.

Considerable progress in recent years has led to the identification of several, but by no means all, of the enzymes and their corresponding genes responsible for AGP glycosylation [14], [15]. In particular, the following enzymes were identified and cloned: two α-1,2-fucosyltransferases (FUT4 and FUT6), one hydroxyproline-*O*-galactosyltransferase (GALT2), one β-1,3-galactosyltransferase (At1g77810), one β-1,6-galactosyltransferase with elongation activity (GALT31A), one β-1,6-galactosyltransferase with branch initiation and branch elongating activities (GALT29A), and three β-1,6-gluronosyltransferases (GlcAT14A, GlcAT14B, GlcAT14C) [16–22]. FUT4 and FUT6 are members of the CAZy GT-37 family; GALT2, At1g77810, and GALT31A are members of the GT-31 family; GALT29A is a member of the GT-29 family; and GlcAT14A, GlcAT14B, GlcAT14C are members of the GT-14 family. In addition, Gille et al. [23] identified a putative AGP β-arabinosyltransferase (RAY1) that is a member of the GT-77 family. This finding, however, is puzzling given that arabinose reportedly only exists as α-linked sugars in AGPs.

AGPs are proposed to play essential roles in a variety of plant growth and development processes, including cell expansion, cell division, reproductive development, somatic embryogenesis, xylem differentiation, abiotic stress responses, and hormone signaling pathways [9], [10], [24–26]. Most of these functions were deduced from analyzing mutants of various AGP genes or by using antibodies or β-Yariv reagent to bind to AGPs and disrupt their function. One complication frequently encountered with AGP gene mutants is that no abnormal phenotype is observed, presumably because of gene redundancy/compensation within the AGP gene family (e.g., there are 85 predicted AGP genes in *Arabibopsis*) [14], [27]. Given that polysaccharides account for approximately 90% of an AGP and largely dictate the molecular surface of an AGP, it is likely that this carbohydrate moiety plays a critical role in AGP function. Thus, mutants in the genes encoding the enzymes for AGP glycosylation may provide a more informative approach to elucidating AGP function.

The hydroxyproline-*O*-galactosyltransferase (Hyp-GALT) that adds the first galactose onto the peptidyl Hyp residues in the AGP core protein is the first committed step in AG polysaccharide addition and represents an ideal control point to investigate the contribution of AG polysaccharides to AGP function. Previously, *GALT2* (*At4g21060*) was demonstrated to encode a Hyp-GALT [17]. Here, another member of GT-31, *GALT5* (*At1g74800*) is shown to encode this same activity. In addition, extensive phenotypic characterization of allelic *galt2* and *galt5* single mutants and *galt2 galt5* double mutants at the biochemical and physiological levels are presented which corroborate the roles of these two enzymes in AG biosynthesis and elucidate the contributions of the AG polysaccharides to AGP function.

## Materials and Methods

### *In silico* analysis of GALT5 and GALT2

Arabidopsis GALT2 and GALT5 predicted protein structure was depicted using Prosite Mydomain Image creator (http://prosite.expasy.org/mydomains/). Transmembrane domain, GALECTIN and GALT domains were predicted using TMHMM server (http://www.cbs.dtu.dk/services/TMHMM/), and Pfam (http://www.sanger.ac.uk/Software/Pfam/) respectively.

### Heterologous expression of *GALT5* in *Pichia pastoris*

The coding region of *GALT5* was obtained from the RIKEN Bioresource center. The open reading frame of *AtGALT5* was amplified with primers with a 5′ restriction site for SacII followed by a 6x His-tag and a 3′ restriction site for XbaI (forward CG**CCGCGG**ATG*CATCATCATCATCATCAC*ATGAAAAAACCCAAATTGTCG and reverse GAGTGTTGTAACATGAGATGA**TCTAGA**). The boldface letters denote the restriction sites, the italic type denotes the His_6_ tag, and the underlined region denotes the translational start site. Amplified products were sequenced, cloned in the shuttle vector pPICZ B as described in Basu et al. (2013). Five individual *Pichia* clones expressing AtGALT5 were selected, and the presence of the gene was confirmed by PCR using genomic DNA isolated from transformants and gene-specific primers. Genomic DNA was isolated from *Pichia* cells as described previously [28]. After confirmation of the clones of *Pichia* harboring *GALT5*, induction for expression of clones expressing GALT5 was performed as described in Basu et al. [17].

### Preparation of *Pichia* microsomes expressing *GALT5* and immunoblot analysis

Microsomal proteins were isolated from the clone five transformed *Pichia* cells as described in Basu et al. [17]. For immunoblot analysis, 5 μg of microsomal protein from *Pichia* transformants was denatured, subjected to 10% SDS-PAGE, and electroblotted onto PVDF Immobilon membranes (Millipore) using the Mini Protean3 system according to manufacturer's recommendations. Blots were probed with an anti-His primary antibody (Clontech) at a 1:10,000 dilution and a secondary goat anti-mouse IgG antibody conjugated to horseradish peroxidase (HRP) (Clontech) at a 1:20,000 dilution. West Femto Maximum Sensitivity Substrate (Thermo Scientific) was used for HRP detection. *Pichia* cell lines transformed with the empty expression vector (pPCIZ B) were used as the negative control (NC). Protein quantification was done using the Bradford reagent (Sigma). Blots were stained with Coomassie Brilliant Blue R-250 (Sigma) following HRP detection to ensure equal loading.

### Galactosyltranferase assay with microsomal preparations from *Pichia* expressing *GALT5*

The standard GALT reaction was performed as described in Basu et al. [17] using detergent permealized microsomes from *Pichia* clone C5 expressing AtGALT5. Two reactions were included as controls, one with no substrate acceptor and one with permeabilized microsomal membranes from the *Pichia* line (X-33) transformed with the empty expression vector (pPICZ B) as NC.

### Purification of Hyp-GALT5 reaction products by reverse-phase HPLC

The GALT reaction products were purified by RP-HPLC as described by Liang et al. [29].

### Analysis of the Hyp-[^14^C]galactoside profile by gel permeation chromatography and high performance anion-exchange chromatography (HPAEC)

Thirty standard GALT reactions were fractionated by RP-HPLC and combined to generate enough ^14^C-radiolabeled product for base hydrolysis and separation on a Biogel P2 column [29]. The radioactive peak eluting at degree of polymerization 4 (DP4) on a Biogel P2 column was analyzed along with a chemically synthesized Hyp-Gal standard by HPAEC on a CarboPac PA-20 column using 5 mM NaOH as the elution buffer to provide additional confirmation of this DP4 peak as Hyp-Gal. *Trans*-4-(β-D-Galactopyranosyloxy)-L-proline (*i*.*e*. the Hyp-Gal standard) was chemically synthesized from commercially available galactopyranosyl bromide and hydroxyproline methyl ester as previously described [17].

### Monosaccharide composition analysis of GALT reaction products by high performance anion-exchange chromatography

Twenty-five standard GALT assays were pooled to generate sufficient ^14^C-products for acid hydrolysis and monosaccharide composition analysis as described by Liang et al. [29] and Basu et al. [17] with minor modifications. The product from total acid hydrolysis was dissolved in deionized water and analyzed on a CarboPac PA20 column (4 × 250 mm; Dionex) in a BioLC system using pulsed amperometric detection (ED50 electrochemical detector; Dionex). The column was equilibrated at a flow rate of 0.5 mL/min with 200 mM NaOH for 10 min, double distilled water for 10 min, and 1mM NaOH for 15 min. The sample was eluted with 1 mM NaOH at a flow rate of 0.5 mL/min.

### Determination of substrate specificity of the GALT5 enzyme activity

A standard GALT assay was performed using 20 μg of various peptide substrate acceptors, (AO)_7_, (AO)_14_, and (containing 7, and 14 repeating dipeptide units, respectively), an extensin peptide (ExtP) containing repetitive SO_4_units, and a (AP)_7_ peptide containing seven AP units as described by Liang et al. [29]. Rhamnogalactan I from potato and rhamnogalactan from soybean (100 μg each) were used as potential pectin substrates. Permeabilized microsomal membranes (250 μg) from the NC *Pichia* line and *Pichia* line expressing His_6_-GALT5 served as the enzyme source in the GALT reactions. For all of the peptide substrate acceptors, the standard GALT assay was performed, and the reaction products were fractionated by RP-HPLC before monitoring incorporation of radiolabeled ^14^C in a liquid scintillation counter (Beckman Coulter LS 6500). For the pectin substrate acceptors, RG (soybean fiber; Megazyme) and RGI (potato; Megazyme), reactions were incubated at room temperature for 2 h, terminated by adding 1 ml of cold 70% ethanol, and precipitated overnight at −20°C. Reaction products were collected by centrifugation at 10,000 × *g* for 10 min, and pellets were resuspended by vortexing followed by ten washes with 1 ml of cold 70% ethanol to remove excess UDP-[^14^C]Gal. The ^14^C-radiolabel incorporation was estimated by resuspending the pellets in 300 μl of water before counting in a liquid scintillation counter.

### Biochemical characterization of *GALT5* enzyme activity

The standard GALT assay was modified for GALT5 characterization using (AO)_7_ peptide as the acceptor substrate. Assay products from each reaction were fractionated by RP-HPLC to measure incorporated ^14^C-radiolabel into acceptor substrates. The optimum pH for GALT5 activity was determined using permeabilized microsomal membranes (250 μg) from the C5 *Pichia* line expressing His_6_-GALT5 dissolved in test buffers at a final concentration of 100 mM. Test buffers included MES-KOH buffer at pH 4, 5, 6, and 7; HEPES-KOH buffer at pH 6, 6.5, 7, 7.5, and 8; Tris-HCl buffer at pH 8, 9, and 10; and CAPS-KOH buffer at pH 9 and 10.

To examine the effect of divalent cations on GALT5 activity, microsomal membranes were extracted with homogenizing buffer lacking divalent ions. MnCl_2_, MgCl_2_, CaCl_2_, CuCl_2_, NiCl_2_, or ZnSO_4_ was added to the GALT assay (at a final concentration of 5 mM) when tested. Two controls were added, one with no ions in the buffer used for resuspending the detergent permealized membrane fraction and the other with EDTA (5 mM) to chelate any residual divalent cations trapped in the membranes. An equal volume of deionized distilled water was added instead of divalent ions in the control reaction.

To analyze the enzyme specificity for nucleotide sugar donors, the standard activity assay was performed with (AO)_7_ as the acceptor substrate and various ^14^C-radiolabeled nucleotide sugar donors (90,000 cpm). The nucleotide sugars tested included UDP-[^14^C]Glc (MP Biomedicals), UDP-[^14^C]Xyl (PerkinElmer Life Sciences), and GDP-[^14^C]Fuc (PerkinElmer Life Sciences). Four separate GALT reactions with no substrate acceptors were performed as controls.

### Transient expression and subcellular localization of GALT5 in *Nicotiana tabacum* leaves

The *GALT5* coding region was subcloned into pEarleyGate 101 plasmid to generate the GALT5:YFP construct by a gateway cloning strategy. The primers used in cloning are listed in S2 Table. *Agrobacterium*-mediated transient expression was performed in the leaves of three to four week-old tobacco plants (*Nicotiana tabacum* cv. Petit Havana) grown at 22–24°C using a bacterial optical density (OD 600) of 0.05 for single infiltrations and 0.025 each for co-infiltrations [30]. The GALT5-YFP construct was co-expressed with either the ER marker GFP-HDEL or the Golgi marker ST-GFP [31] to ascribe subcellular localization. The ER and Golgi markers are cloned into pVKH18-EN6 plasmid vector. Transformed plants were incubated under normal growth conditions and sampled daily for 2–5 days post-infiltration. Leaf epidermal sections were imaged using an upright Zeiss LSM 510 META laser scanning microscope (Jena, Germany), using a 40 X oil immersion lens and an argon laser. For imaging the expression of YFP constructs, the excitation line was 514 nm, and emission data were collected at 535–590 nm, whereas for GFP constructs, the excitation line was 458 nm and the emission data were collected at 505–530 nm. Singly infiltrated controls were analyzed to optimize gain and pinhole settings for each channel and to exclude any bleed through fluorescence between channels. Post-acquisition image processing was done using the ZEN lite 2012 image analysis software (Blue Edition; Carl Zeiss).

### Plant material and genetic analysis

The Columbia (Col-0) ecotype of Arabidopsis thaliana was used in this study. Two T-DNA insertional lines for *At1g74800*-*GALT5* (*galt5-1* SALK_105404 and *galt5-2* SALK_115741) and *At4g21060*-*GALT2* (*galt2-1* SALK_117233 and *galt2-2* SALK_141126) were selected using the SIGnaL database (http://signal.salk.edu/) and were obtained from the ABRC (Arabidopsis Biological Research Centre) (http://abrc.osu.edu/). Other mutants including *fei1* (SALK_080073), *fei2-1*, and *sos5-2* (SALK_125874) were provided by Dr. Joseph Keiber. Arabidopsis plants used in this study were germinated after 4 days of stratification in the dark at 4°C, and grown on soil at 22°C with 60% relative humidity. Plants were grown under long-day conditions (16 h photoperiod and 8 h dark, 120 μmol m ^-2^ s ^-1^ of fluorescent light).

Genomic DNA was isolated from *galt5-1*, *galt5-2*, *galt2-1*, *galt2-2* and *galt2 galt5* mutant leaves and subsequent PCR analysis was carried out using Extract-N-Amp Plant Kits (Sigma-Aldrich). The primer sequences used in PCR analysis were obtained from the T-DNA Primer Design Tool provided by the Salk Institute Genomics Analysis Laboratory (http://signal.salk.edu/tdnaprimers.2.html) in conjunction with the gene specific left and right primers (S2 Table). PCR products were purified by gel extraction with QIAquick Gel Extraction Kit and sequenced by the Ohio University Genomics Facility. To confirm homozygous plants at the transcript level, RNA was extracted, reverse transcribed, and analyzed by PCR using RT primers. RNA was isolated using a Qiagen RNeasy plant mini kit followed by DNase I digestion using Qiagen RNase free DNase set to remove traces of DNA. Qiagen One-Step RT-PCR kit was used for first-strand synthesis and subsequent PCR steps (primers are listed in S2 Table).

For quantitative real-time PCR (qPCR), the cDNAs were amplified using Brilliant II SYBR Green QRT-PCR Master Mix with ROX (Agilent Technologies, La Jolla, CA, USA) in an MX3000P real-time PCR instrument (Agilent Technologies). PCR was optimized and reactions were performed in triplicate. The transcript level was standardized based on cDNA amplification of *Ubiquitin 10* (*At4g05320*) RNA as a reference.

### Isolation of Golgi-enriched plant microsomal membranes

Plant microsomal membranes were extracted according to Liang et al. [29] with minor modifications. Eight grams of leaf tissue from 14-d-old wild type, *galt2-1*, *galt2-2*, *galt5-1*, *galt5-2* and *galt2 galt5* mutant plants were ground in liquid nitrogen followed by resuspension in 8 ml extraction buffer (0.1 M HEPES-KOH, pH 7, 0.4 M Sucrose, 1 mM dithiothreitol, 5 mM MgCl_2_, 5 mM MnCl_2_, 1 mM phenylmethylsulfonyl fluoride, and one tablet of Roche EDTA-free complete protease inhibitor cocktail and 100 μL RPI plant protease inhibitor VI). The homogenate was filtered through two layers of miracloth, and the filtrate was centrifuged at 3,000 x g for 20 min. The resulting supernatant was layered over a 1.8 M Sucrose cushion buffer and centrifuged at 100,000 x g for 60 min. The uppermost layer was discarded without disturbing the membrane containing interphase layer. A discontinuous sucrose gradient was implemented by sequentially layering 1.1 and 0.25 M sucrose solutions onto the interphase layer and centrifuging at 10,000 x g for 60 min. The microsomal membranes enriched at the 0.25/1.1 M sucrose interphase were collected and pelleted by another centrifugation at 100,000 x g for 30 min. The pellet was resuspended in 50 μL extraction buffer and stored at −80°C until use. A 1% Triton-X 100 permealized membrane fraction was used to perform GALT reactions using [AO]_7_ as the peptide substrate acceptor and UDP-[^14^C]Gal as the sugar donor.

### Extraction of AGPs and AGP profiling by HPLC

AGPs were extracted from 14-d-old WT, *galt2-1*, *galt2-2*, *galt5-1*, *galt5-2*, and *galt2 galt5* mutant plants as described in Schultz et al. [32]. Five grams of plant material was used for each of the lines. Quantification of AGPs was done following the method of Gao et al. [33], and β-Gal Yariv reagent was prepared as described in Yariv et al. [34].

AGP profiling was conducted as described by Youl et al. [35] with modifications. AGPs were obtained from eight grams of 14-d-old WT and mutant plants, precipitated by β-Gal Yariv reagent and dissolved in 1 mL of deionized water before applying 100 μl onto a polymeric reverse-phase column (PRP-1, 5 μm, 4.1 × 150 mm; Hamilton) equilibrated with buffer A (0.1% trifluoroacetic acid). Fifty microgram of [AO]_7_ was used as a control to monitor the retention time of a pure AGP peptide. Samples were eluted from the column following a linear gradient with solvent B (0.1% trifluoroacetic acid in 80% acetonitrile): 0 to 30% solvent B in 30 min, then 30 to 100% in 30 min at a flow rate of 0.5 mL/min. Chromatography was monitored by absorption at 215 and 280 nm.

### *In vitro* pollen germination assay

Flowers collected from WT, *galt2-1*, *galt2-2*, *galt5-1*, *galt5-2* plants 1 to 2 weeks after bolting were used for the examination of pollen tube phenotypes. Individual open flowers were germinated *in vitro* as described by Boavida and McCormick [36] on solid germination medium (0.01% H_3_BO_3_, 1 mM MgSO_4_, 5 mM KCl, 5 mM CaCl_2_, 10% sucrose, and 1.5% low-melting agarose, pH 7.5 and 30 μM β-Gal Yariv reagent or 30 μM α-Gal Yariv reagent) at 22°C and 100% humidity in the dark. Pollen tube germination rates were computed by dividing the total number of germinated tubes by the number of grains. Images and measurements of pollen tubes were done at either at 40X or 20X magnification in a Nikon microscope coupled with a SPOT RT color CCD camera and SPOT analysis software.

### Germination assays

Seeds of wild type, *galt2-1*, *galt2-2*, *galt5-1* and *galt5-2* were surface-sterilized by washing in a 95% ethanol solution for 5 min followed by a 5 min wash in a 30% bleach with 0.1% Tween 20 solution and then rinsed seven times with sterile water. The seeds were sown on 1X MS nutrient medium containing 1% sucrose and 0.6% agar. For stratification treatment, seeds were stratified at 4°C in the dark for 3 d. The germination rate was scored by counting the number of germinated seeds after 5 d. Experiments were done in triplicate with 50 seeds for each experiment and genotype. Only seed batches that had been harvested and stored at the same time and under the same conditions were used. For each experiment, samples from four genotypes (WT, two allelic single mutants and the double mutant) were placed side by side on the same plate. Various concentrations of NaCl, KCl, LiCl, CsCl, mannitol or 50 μM α-Gal Yariv reagent or 50 μM β-Gal Yariv reagent were added to the MS media. Germination (i.e., emergence of radicles) was measured under a compound microscope at intervals of 12 h for 5 d. Radicle length was measured by Motic Image version 3.2. Three replicate plates were used for each treatment to ensure reproducibility of data.

### Root growth measurements

For monitoring root growth in response to Yariv reagent, wild type, *galt2-1*, *galt2-2*, *galt5-1*, *galt5-2* and *galt2 galt5* were grown on MS plates for 7 d before they were transferred to MS plates supplemented with 50 μM α-Gal Yariv reagent or 50 μM β-Gal Yariv reagent. For seedling growth in salt, 7-d-old seedlings of wild-type, *galt2-1*, *galt2-2*, *galt5-1*, *galt5-2* and *galt2 galt5* plants were transferred to MS medium containing 1% agar and 100 mM or 150 mM NaCl. Root length was determined on low-magnification (×10) digital images captured using a CCD camera and image analysis freeware (ImageJ; http://rsb.info.nih.gov/ij/). For analysis of salt hypersensitivity of the mutant plants, root growth was monitored using a root bending assay [37] and images were taken under Nikon SMZ1500 stereomicroscope coupled with a CCD Infinity 2 camera and analysis software.

### Aberrant root hair morphology

Root hair length from 8-d-old plants grown on agar plates was determined on low-magnification (×10) digital images captured using a CCD camera and image analysis freeware (ImageJ; http://rsb.info.nih.gov/ij/). To ensure comparable results, the area 3 to 5 mm behind the root tip was analyzed. Plants grown on agar plates were carefully removed in ∼100 μL of half-strength MS medium on microscope slides for analysis. Quantification data are the means of 50 to 75 values representing 15 root hairs each of 20 to 35 individual plants measured.

### Seed staining and visualization

Seeds of all the indicated genotypes were prehydrated in water and stained either with 0.01% ruthenium red or calcofluor white (25 μg/ml of fluorescent brightener). In both cases, staining was performed as described by Willats et al. [38] and Harpaz-Saad et al. [39]. Imaging was done using a Zeiss LSM 510 confocal microscope.

### AGP specific monoclonal antibodies

Four AGP specific monoclonal antibodies, JIM4, JIM8, JIM13 and MAC207, were obtained from CarboSource Services; http://www.ccrc.uga.edu/~carbosource/CSS_home.html) and used as primary antibodies for detection of AGP epitopes. Goat anti-rat secondary antibody conjugated to fluorescein isothiocyanate (FITC) was used as secondary antibody. Root hairs, pollen tubes and seeds treated with secondary antibody only were used as negative controls. Images were examined with a Zeiss LSM 510 laser scanning confocal microscope equipped with an argon-ion laser, using single wavelength excitation at 488 nm and detection of FITC signals between 505 and 530 nm. Confocal parameters for each antibody treatment were preserved across genotypes. Z-stack sections of the images were taken, and three-dimensional projections from these stacks were used for the final images using LSM Software ZEN 2011.

### Immunofluorescence detection of AGPs epitopes in root hairs, pollen tubes and seeds

Ten-day-old WT and *galt2galt5* seedlings grown on MS plates were used for immuno-staining of AGP epitopes according to the method described by Sauer et al. [40]. Briefly, seedlings were harvested in 1X MS liquid media followed by fixation in stabilizing buffer (SB) prepared in 1X MS media containing 50 mM PIPES buffer, 5 mM MgSO_4_, 5 mM EGTA pH 7.0 with 4% paraformaldehyde at 4°C overnight. After extensively washing seedlings with SB without 4% paraformaldehyde, they were incubated for 60 min at room temperature in 1X MS media containing 3% IGEPAL followed by incubation for 1h with 3% BSA with 0.02% sodium azide in 1X MS. Seedlings were incubated with the primary antibody (1:25 dilution) overnight at 4°C in the dark, followed by extensive rinsing and incubation with secondary antibody at a 1:50 dilution in 1X MS media for 5 h at room temperature. Finally, seedlings were washed in 1X MS media and mounted in 25% glycerol in 1X MS media.

Immunolocalization of AGPs in WT and *galt2galt5* pollen tubes were performed according to the method described by Dardelle et al. [41]. Briefly, pollen tubes were germinated in germination media (GM) containing 5 mM CaCl_2_· 2H_2_O, 0.01% (w/v) H_3_BO_3_, 5 mM KCl, 1 mM MgSO_4_· 7H_2_O, and 10% (w/v) Suc, pH 7.5 for 16 h at room temperature. Upon germination, they were mixed (v/v) with a fixation medium containing 100 mM PIPES buffer, pH 6.9, 4 mM MgSO_4_· 7H_2_O, 4mM EGTA, 10% (w/v) Suc, and 5% (w/v) formaldehyde and incubated for 90 min at room temperature. Pollen tubes were rinsed three times by centrifugation at 3,200 g for 6 min with 50 mM PIPES buffer, pH 6.9, 2 mM MgSO_4_· 7H_2_O, and 2 mM EGTA and three times with phosphate-buffered saline (100 mM potassium phosphate, 138 mM NaCl, and 2.7 mM KCl, pH 7.4). Pollen tubes were incubated overnight at 4°C in the dark with primary antibodies diluted 1:10 with phosphate-buffered saline, rinsed, and incubated with secondary antibody diluted 1:50 for 3 h at room temperature.

Whole-seed immunolabeling was conducted according to the method described by Harpaz-Saad et al.[39], using seeds shaken in water before immunolabeling.

## Results

### At1g74800 (*GALT5*) encodes a putative galactosyltransferase

GALT5 and GALT2 proteins are members of the diverse GT-31 family in the CAZy database [41]. In plants, GT-31 includes three clades; one with proteins having only a catalytic GALT domain, another with proteins containing both a galactosyltransferase (GALT) domain and a GALECTIN domain, and the third with proteins having a domain of unknown function [42]. In Arabidopsis, 14 proteins have only the GALT domain, 6 proteins contain both domains, and 13 proteins have a domain of unknown function [18]. Four of these Arabidopsis GT-31 family members have been characterized. At1g26810 (GALT1) was identified as a β-(1,3)-GALT involved in biosynthesis of a Lewis a epitope on N-linked glycans [43]. At1g77810 was reported to be a β-(1,3)-GALT that catalyzes transfer of galactose (Gal) to an *O*-methylated Gal-β-(1,3)-Gal disaccharide, which mimics a partial structure of AGP side chains [18]. At4g21060 (GALT2) was identified as a Hyp-GALT specific for AGPs [17]. Finally, At1g32390 (GALT31A) was shown to elongate β–1,6-galactan side chains on AGPs [19]. Given that glycosyltransferases containing a lectin domain are involved in catalyzing the first step of *O*-glycosylation of animal glycoprotein mucins, it was hypothesized that plant GALTs containing analogous lectin domains may also function in initiating *O*-glycosylation of AGPs. Thus, we focused on functional characterization of such GALT genes containing a GALECTIN domain and here present our findings on GALT5. The GALT5 open reading frame is 2019 bp and corresponds to a protein of 672 amino acids, with a calculated molecular mass of 77.3 kD. The predicted protein structures and alignment of GALT2 and GALT5 are depicted in S1 Fig Both proteins are predicted to be type II membrane proteins with N-terminal transmembrane domains. Thus, we hypothesized that GALT5 protein functions as an AGP-specific Hyp-GALT.

### Heterologous expression of GALT5 in *Pichia* cells

Microsomal proteins from five independent recombinant *Pichia* lines expressing His tagged GALT5 were examined by immunoblotting with antibodies directed against the 6x His tag. All five GALT5 recombinant lines had the expected 77 kD protein band that reacted with the 6x His antibody (S2A Fig). A non-specific, smaller protein band (50 kD) was also detected in these recombinant lines. Transformed *Pichia* cells with the empty expression vector served as a negative control (NC) and lacked the recombinant 77 kD protein band, but contained the 50 kD protein band.

### Heterologously expressed GALT5 demonstrates Hyp-GALT activity

An *in vitro* GALT assay developed by Liang et al. [29] was used to test for activity of the recombinant GALT5 expressed in *Pichia* cells. GALT assay components included detergent-permeablized microsomal membranes from the transformed *Pichia* cell lines expressing GALT5 protein as the enzyme source, UDP-[^14^C]Gal as the sugar donor and one of two AGP peptide analogs (d[AO]_51_ and [AO]_7_) as the substrate acceptor. The amount of GALT activity varied in the five recombinant cloned cell lines (C1 to C5) of *Pichia* based on the rate of [^14^C]Gal incorporation using the [AO]_7_ substrate acceptor, but all were significantly higher than *Pichia* cells transformed with the vector alone, which served as a negative control (NC) (S2B Fig).

### Characterization of the GALT5 assay products by reverse-phase HPLC analysis

**Fig 1:**
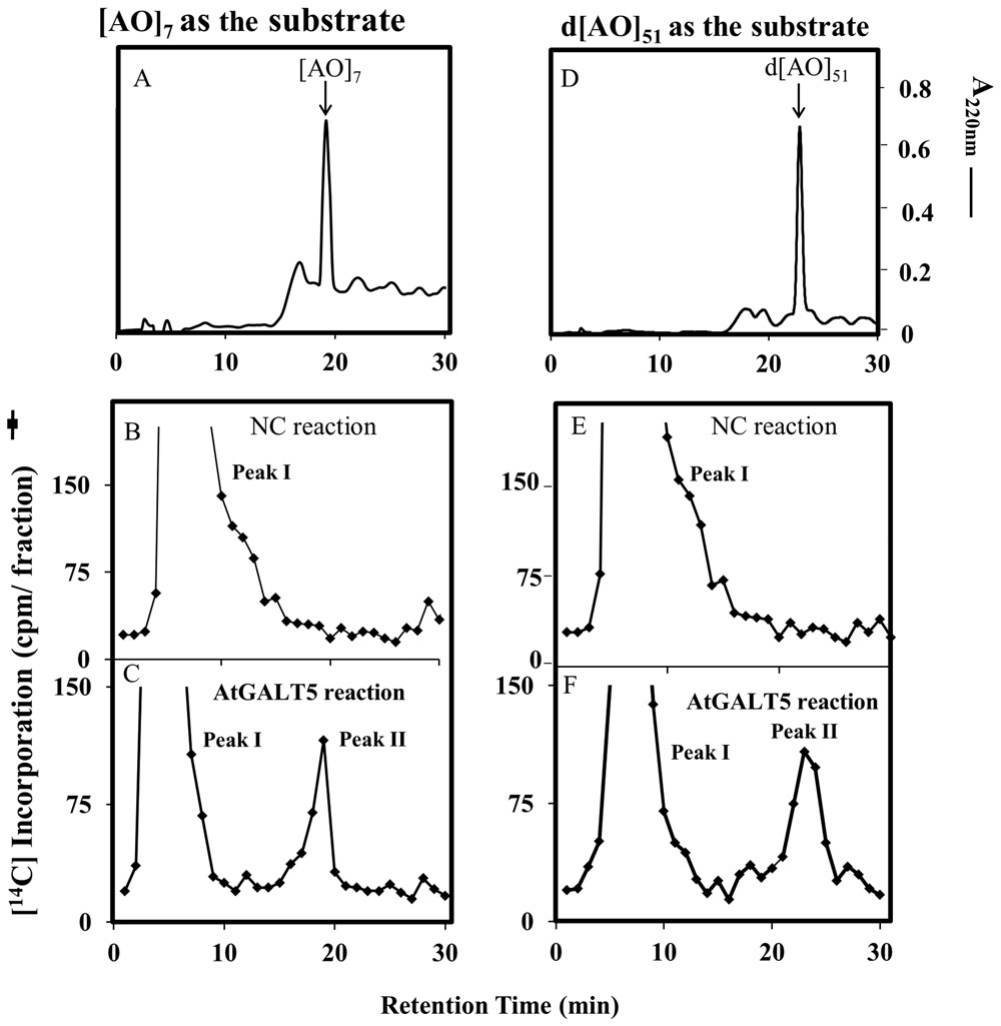
RP-HPLC fractionation of the [AO]_7_:GALT5 reaction products on a PRP-1 reverse-phase column. Acceptor substrate alone (A and D), GALT reaction with microsomal membranes from the NC *Pichia* line transformed with the empty expression vector (B and E) and the GALT reaction with microsomal membranes from the transgenic *Pichia* C5 line (C and F) were fractionated by RP-HPLC using identical elution conditions. Radioactive Peak II coeluted with the [AO]_7_ and d[AO]_51_ acceptor substrates in the GALT5 reaction and was used for subsequent product analysis.

*Pichia* transformants expressing GALT5 were further analyzed for Hyp-GALT activity using two substrate acceptors: [AO]_7_, a synthetic AGP peptide and d[AO]_51_, a transgenically expressed and chemically deglycosylated AGP analog. Incorporation of [^14^C]Gal from UDP-[^14^C]Gal onto the two substrate acceptors was observed by HPLC fractionation (Fig 1C and 1F) and by comparison to the non-radioactive [AO]_7_ and d[AO]_51_ substrate acceptor peaks (Fig 1A and 1D). Two [^14^C]-radioactive peaks were detected, of which peak II has the same retention time as their respective substrate acceptors ([AO]_7_ and d[AO]_51_) (Fig 1C and 1F). The identity of peak I is not known; it may represent free [^14^C]Gal released by an endogenous galactosidase [29] or be composed of oligosaccharides with [^14^C]Gal incorporated into endogenous sugar acceptors as suggested previously [44]. Peak I was also present in previous studies with plant (Arabidopsis and tobacco BY2) microsomes [29]. Microsomal preparations from a *Pichia* cell line transformed with the empty expression vector were used as negative controls (NC) (Fig 1B and 1E). In summary, HPLC fractionation provided evidence for incorporation of the [^14^C]radiolabel from UDP-[^14^C]Gal onto the [AO]_7_ and d[AO]_51_ acceptors, and the [AO]_7_:GALT5 reaction product was subjected to further biochemical characterization.

### Product characterization by acid and base hydrolysis indicates GALT5 transfers Gal to Hyp residues

To confirm that the [^14^C]radiolabel remained associated with Gal, RP-HPLC fractions containing the [^14^C]radiolabeled [AO]_7_:GALT5 reaction products were pooled and subjected to total acid hydrolysis. The resulting acid hydrolyzed [^14^C]radiolabeled monosaccharide was fractionated by HPAEC and showed that the [^14^C]label co-eluted with Gal, thereby confirming incorporation of [^14^C]Gal onto the [AO]_7_ peptide (S3 Fig).

**Fig 2:**
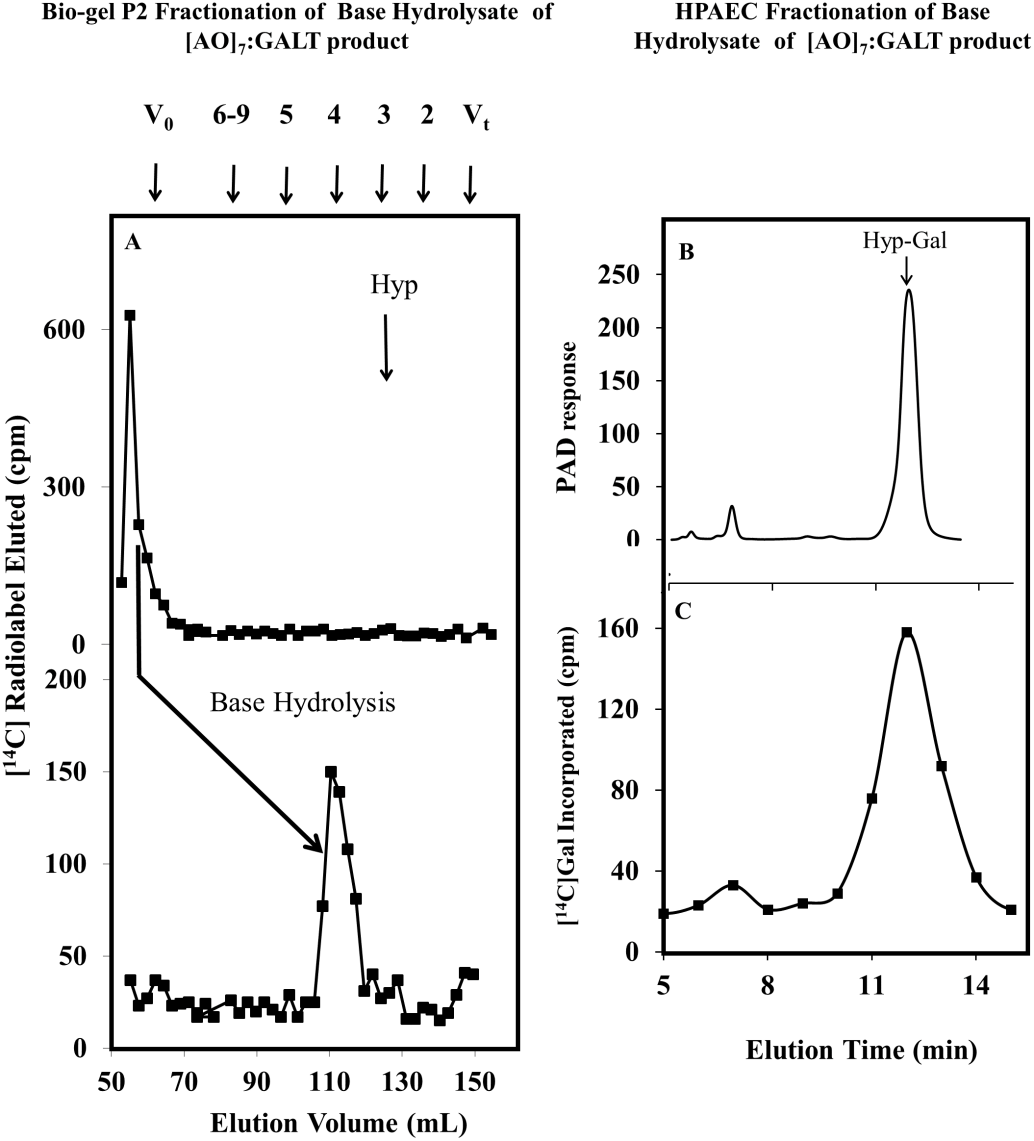
Bio-gel P2 fractionation of the RP-HPLC purified [AO]_7_:GALT5 reaction product and High-Performance Anion-Exchange Chromatography (HPAEC) of the resulting base hydrolysis product. (A) Bio-gel P2 fractionation of the RP-HPLC purified [AO]_7_:GALT5 reaction product before and after base hydroylysis. Permeablized microsomal membranes from the *Pichia* C5 line expressing 6x His-GALT5 served as the enzyme source in the [AO]_7_:GALT5 reaction. Elution profiles of the reaction product before and after base hydrolysis are shown. The column was calibrated with high-*M*_*r*_ dextran (V_0_), galactose (V_t_), xylo-oligosaccharides with degree of polymerization (DP) 2 to 5 and xyloglucan-oligosaccharides (DP6-9); their elution positions are indicated with arrows at the top of the figure. The elution position of free Hyp amino acid (corresponding to DP3) is shown with an arrow in the panel. Base hydrolysis produces a radioactive peak eluting at DP4, which corresponds to Hyp-Gal. (B) HPAEC profile of a chemically synthesized Hyp-Gal standard detected as a PAD response. (C) The radioactive peak eluting at DP4 coelutes with the chemically synthesized Hyp-Gal standard following HPAEC. Both the Hyp-Gal standard and the radioactive peak eluting at DP4 were fractionated in 5 mM NaOH elution buffer on a CarboPac PA-20 column.

In another set of experiments, base hydrolysis was used to confirm that the [^14^C]Gal residues were added to Hyp residues and to examine the extent of galactosylation of the [AO]_7_ peptide acceptor. Base hydrolysis degrades peptide bonds, but keeps Hyp-glycosidic bonds intact [45]. The intact [^14^C]radiolabeled [AO]_7_ peptide product eluted in the void volume (V_0_) on the P2 column, whereas the base hydrolysate of this product eluted at DP4 (Fig 2A). Given that Hyp residues alone elute as a DP3 sugar on a P2 column, it was concluded that GALT5 catalyzes the addition of one Gal onto the [AO]_7_ peptide, consistent with our previous work [17]. Further confirmation of this conclusion was provided by fractionation of the base hydrolysate on a CarboPac PA-20 column and observing that the [^14^C]radiolabel co-eluted with an authentic Hyp-Gal standard (Fig 2B and 2C).

### GALT5 is specific for AGPs

**Fig 3:**
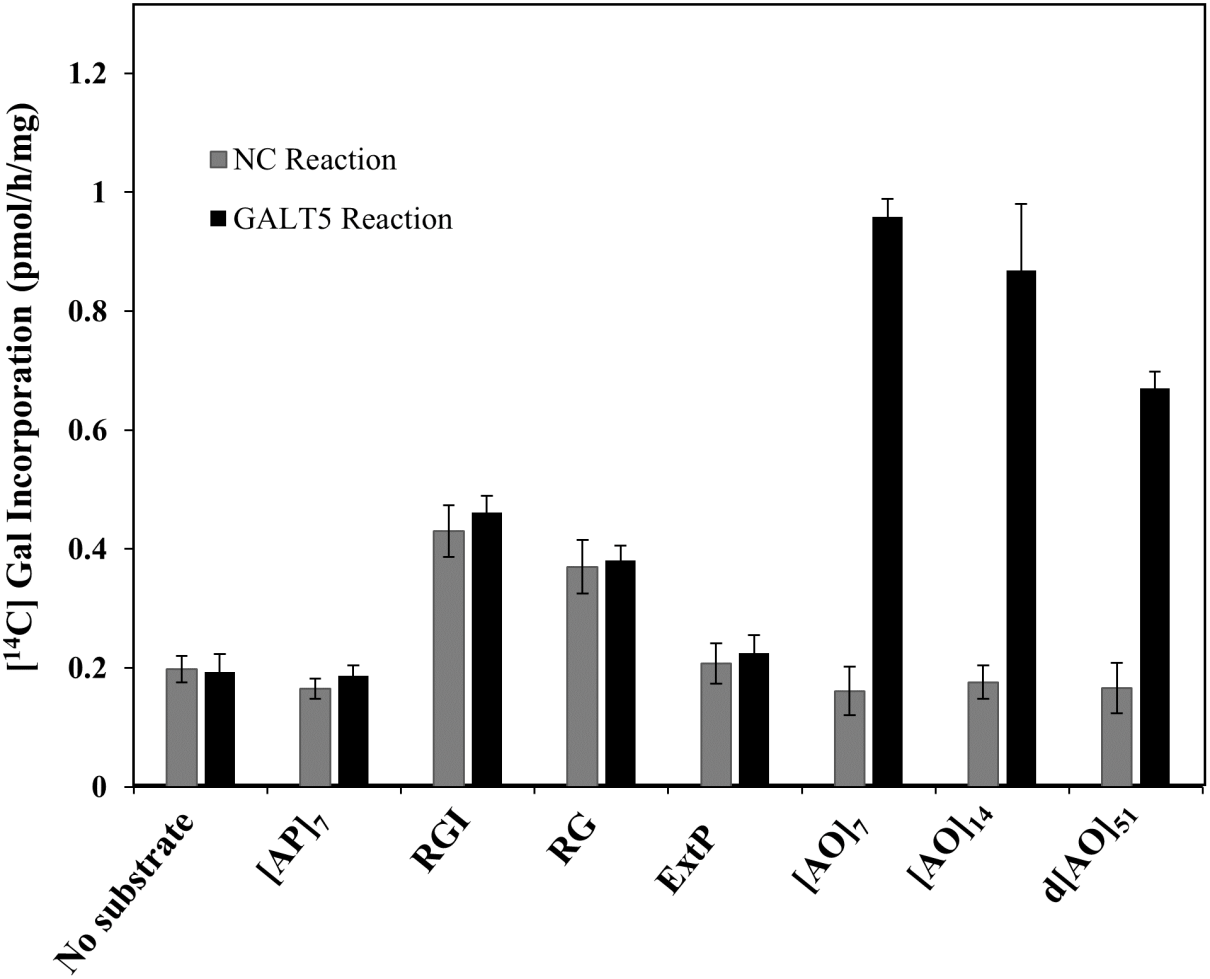
Effect of various peptide and polysaccharide acceptor substrates on incorporation of [^14^C]radiolabeled galactose. Permeablized microsomal membranes from the NC *Pichia* line transformed with the empty expression vector and the C5 *Pichia* line expressing 6x His-GALT5 served as the enzyme source in the GALT reactions. [AO]_7_, [AO]_14_, and d[AO]_51_ contain 7, 14, and 51 [AO] units, respectively. A chemically synthesized extensin peptide (ExtP) contains repetitive SO_4_ units. [AP]_7_ contains 7 [AP] units. Rhamnogalactan I (RGI) from potato and RG from soybean represent pectin polymer substrates. Enzyme reactions using UDP-[^14^C]Gal as the sugar donor were done in triplicate and mean values ±SE are presented.

Various substrates that might act as potential substrate acceptors for a GALT were tested to investigate GALT5 enzyme specificity. Namely, [AO]_7_, [AO]_14_, and d[AO]_51_, consisting of non-contiguous peptidyl Hyp residues, were used to examine AGP peptide sequences of various lengths. [AP]_7_, consisting of alternating Ala and Pro residues, was used to test the requirement of peptidyl Hyp for galactosylation. ExtP, a chemically synthesized extensin peptide consisting of contiguous peptidyl Hyp residues, was used to test whether contiguous peptidyl Hyp residues act as potential acceptors. Two pectic polysaccharides, RGI from potato and RG from soybean fiber, were also used as potential substrates acceptors. All the non—AGP substrate acceptors, including [AP]_7_, failed to incorporate [^14^C]Gal, indicating the GALT5 activity was specific for AGP sequences containing non-contiguous peptidyl Hyp. It was also observed that the incorporation of the [^14^C]radiolabel decreased with increasing lengths of the [AO] acceptor substrates (Fig 3).

### Biochemical characteristics of the GALT5 enzyme

To determine the preference of nucleotide sugar donors, the standard GALT assay was performed with other potential sugar nucleotides including UDP-[^14^C]Glc, UDP-[^14^C]Xyl, and GDP-[^14^C]Fuc in the presence and absence of the [AO]_7_ peptide acceptor. Hyp-GALT activity was only detected with UDP-[^14^C]Gal as the sugar donor (S4A Fig). The effects of pH and divalent cations on the GALT assay catalyzed by GALT5 were also determined. The [AO]_7_:GALT5 activity had a pH optimum of 6.5 with a HEPES-KOH buffer, which is consistent with the lumen of Golgi vesicles where the enzyme is predicted to be localized (S4B Fig). Mg^2+^ followed by Mn^2+^ significantly enhanced GALT5 activity, whereas the presence of Ca^2+^, Cu^2+^, Zn^2+^, and Ni^2+^ had inhibitory effects to different extents (S4C Fig).

### GALT5 is localized to the Golgi

**Fig 4:**
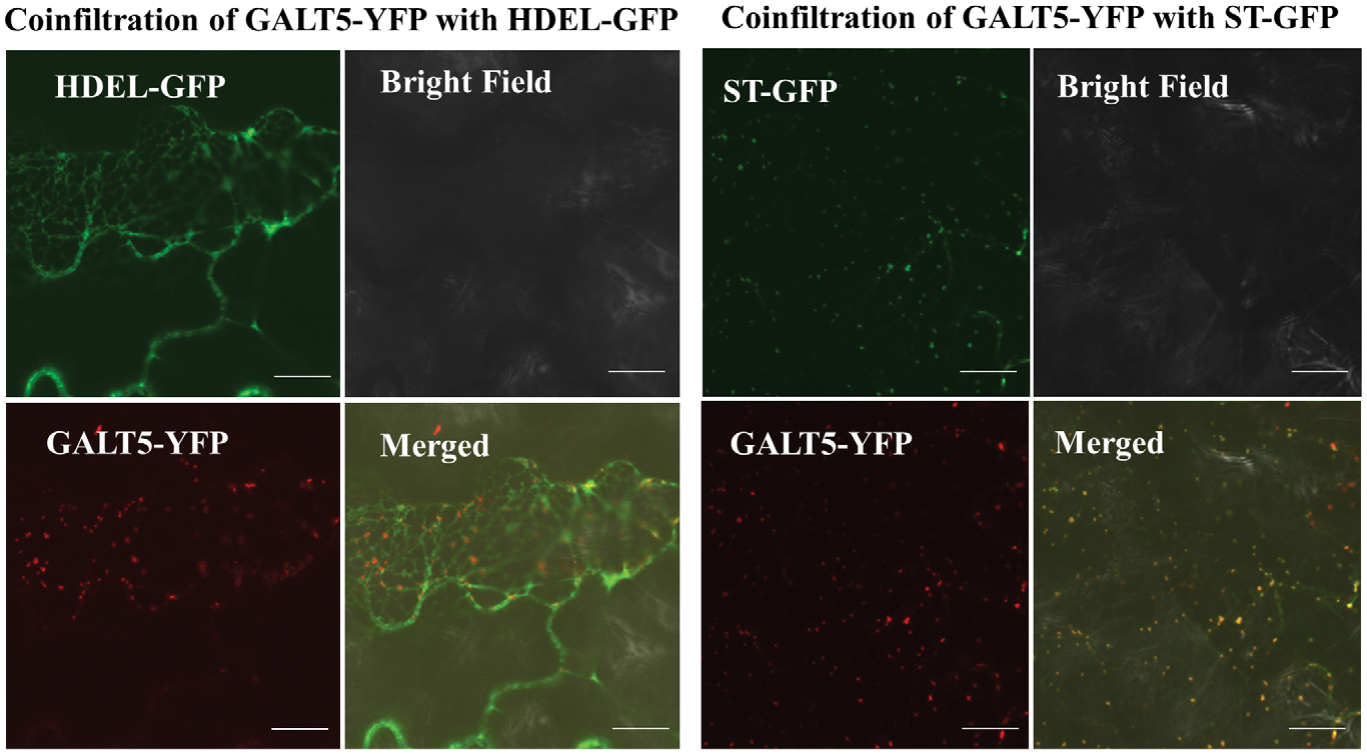
Subcellular localization of GALT5 in tobacco leaf epidermal cells observed 5 days after infiltration. Transiently expressed GALT5-YFP co-localized with sialyltransferase (ST)-GFP fusion protein (a Golgi marker) as well as with HDEL-GFP fusion protein (an ER marker). These constructs were examined by laser-scanning confocal microscopy under fluorescent and white light, and the fluorescent images were merged to observe co-localization. Bar = 10μm.

To establish the subcellular localization of GALT5, live-cell confocal imaging of fluorescently tagged GALT5 protein was performed. A GALT5-YFP fusion was constructed and transiently co-expressed with a Golgi marker protein, sialyltransferase (ST)-GFP, or an ER marker, HDEL-GFP, in tobacco leaves. Upon co-infiltration with the Golgi marker, GALT5-YFP was observed to co-localize with the Golgi marker as discrete punctate structures typical of a Golgi-localized staining pattern (Fig 4). Whereas upon co-infiltration with the ER marker, GALT5-YFP was not observed in the characteristic reticulate structures typical of ER localization (Fig 4). GALT5 was also examined using multiple subcellular localization prediction programs (TargetP, http://www.cbs.dtu.dk/services/TargetP/) and Golgi Predictor http://ccb.imb.uq.edu.au/golgi/) and the TMHMM server (http://www.cbs.dtu.dk/services/TMHMM/) [46] for the prediction of transmembrane domains (TMD). Based on these analyses and consistent with the live cell imaging data, GALT5 is targeted to the secretory pathway and has a single N-terminal TMD (S1 Fig).

### Isolation of T-DNA insertion alleles for the *GALT2 and GALT5* genes

**Fig 5:**
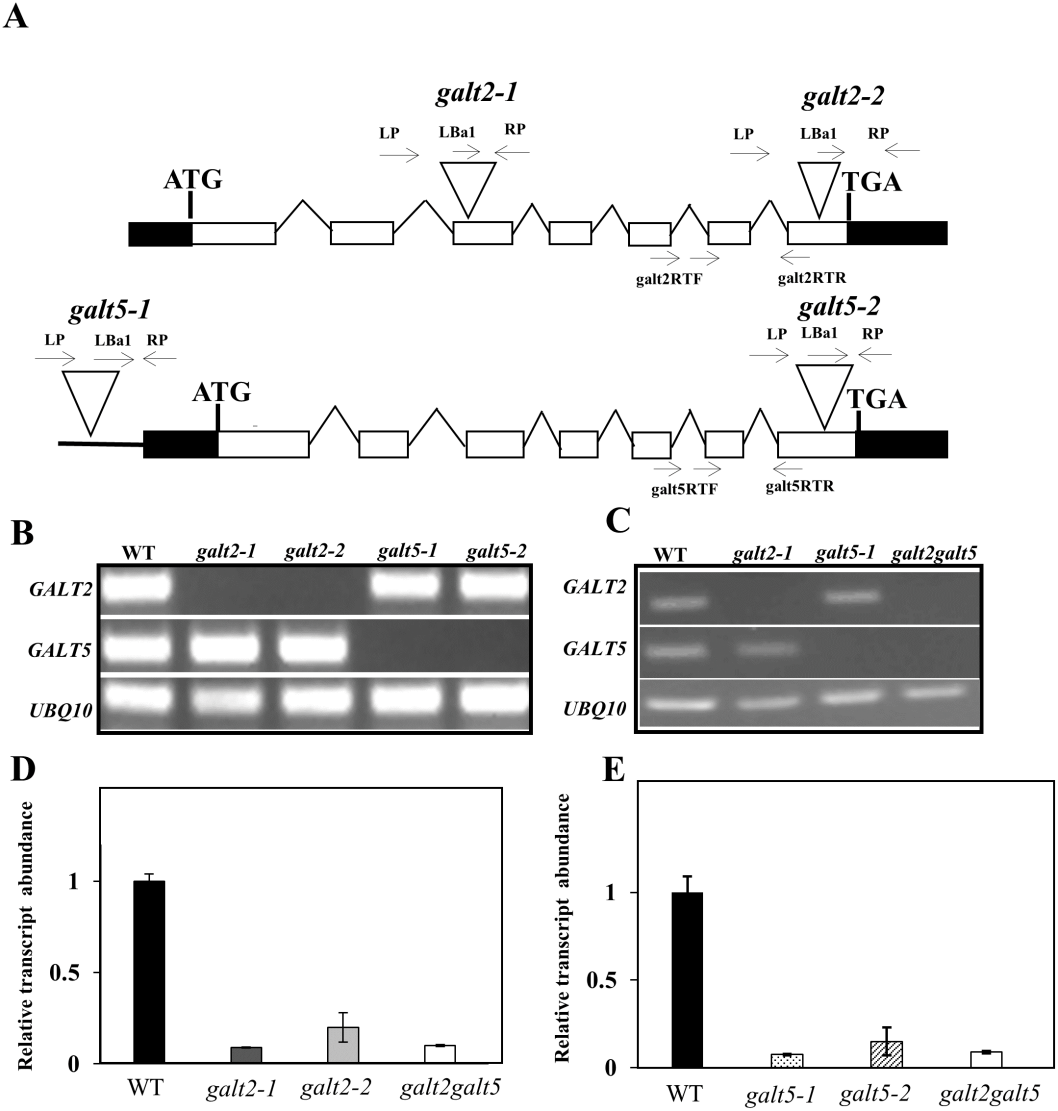
Molecular characterization of *galt* single and double mutants. (A) *GALT2* and *GALT5* gene structure and T-DNA insertion sites in *galt2-1*, *galt2-2*, *galt5*-1 and *galt5-2* mutants. The intron-exon structures of *GALT2* and *AGALT5* are indicated (introns are drawn as lines and exons as rectangles, with white rectangles representing coding sequences and black rectangles representing UTRs). Sites of T-DNA insertions in *galt2* and *galt5* are marked (triangles) as are the locations of primer sequences (arrows) used for PCR screening. (B) RT-PCR analysis of transcripts from rosette leaves of 14-d-old wild type (Col-0), the allelic homozygous *galt2* and *galt5* mutant lines. Arrows indicate the position of primers used for RT-PCR analysis of transcript levels. *UBQ10* primers were used as internal controls. (C) RT-PCR analysis of transcripts from rosette leaves of 14-d-old wild type (Col-0), the homozygous *galt2* and *galt5* mutant lines used for producing the double mutant, and the *galt2 galt5* double mutant. (D) and (E) Quantitative RT-PCR analysis to detect *GALT2* and *GALT5* transcript abundance in the *galt* mutants. Total RNA was isolated from rosette leaves of 14-d-old wild type, *galt2-1*, *galt2-2*, *galt5-1*, *galt5-2*, and *galt2 galt5* plants. *UBQ10* primers were used as controls. Data were normalized to the level of wild type *GALT2* expression in panel D and wild type *GALT5* expression in panel E, which was set to 1 arbitrary unit (a.u.) in each case. Means ± SE of three biological replicates (n = 3) are shown.

To elucidate the *in vivo* functions of *GALT2* and *GALT5* in *Arabidopsis*, a reverse genetic approach was adopted. Two independent mutant alleles were isolated for each of the genes, *galt2*-*1* and *galt2-2* for *GALT2* and *galt5-1* and *galt5-2* for *GALT5*. Homozygous lines were identified by PCR analysis and T-DNA insertion sites were confirmed by sequencing (Fig 5A). Testing for genetic redundancy was addressed by crossing *galt2* and *galt5* single mutants and using PCR to screen for *galt2 galt5* double mutants in the resulting F2 generation. RT-PCR analysis showed that the *GALT2* transcript was absent in both *galt2* allelic mutants as well as in the double mutant and that the *GALT5* transcript was absent in both *galt5* allelic mutants as well as in the double mutant (Fig 5B and 5C). The qPCR analysis corroborated these findings and confirmed the identification of allelic knock-out *galt2*, *galt5* single mutants as well as *galt2 galt5* double mutants (Fig 5D and 5E).

### *GALT2 and GALT5* have overlapping but distinct expression patterns

**Fig 6:**
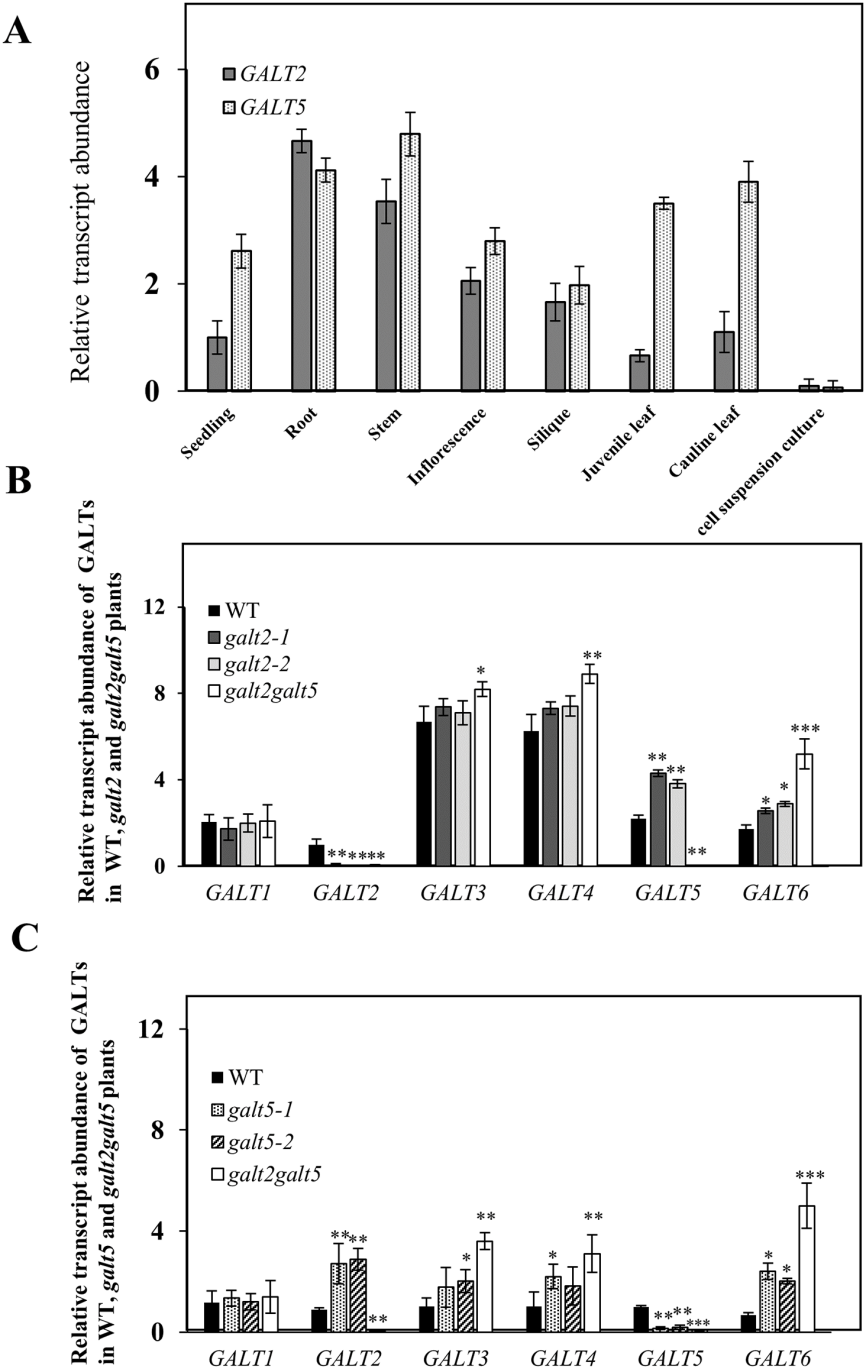
Organ-specific expression of *GALT2* and GALT5 and gene compensation in *galt2*, *galt5* and *galt2 galt5* mutants observed by quantitative RT-PCR analysis. (A) Organ-specific relative expression of *GALT2* and *GALT5* genes. qPCR was performed with total RNA samples from roots, stem, inflorescence, silique, seedling, cell culture, cauline leaves, and juvenile rosette leaves. The averages of three biological replicates are shown. The y axis shows x-fold expression with respect to the lowest encountered value of *GALT2* expression in seedling equal to one arbitrary unit (a.u.). (B) Functional compensation of *galt2* and *galt2 galt5* as revealed by qPCR analysis. Data were normalized to the level of *GALT2* expression in seedlings of wild type, which was set to 1 arbitrary unit (a.u.). RNA was isolated from 14-d-old seedlings. (C) Functional compensation of *galt5* and *galt2 galt5* as revealed by qPCR analysis. The expression values were normalized to the level of *GALT5* expression in seedlings of wild type, which was set to 1 arbitrary unit (a.u.). *UBQ10* was used as an internal control for all the qPCR experiments. The asterisks indicate significant differences in expression of transcripts of the six *GALTs* tested compared with wild type according to a Student's t test (*, P < 0.05; **, P < 0.01, ***, P<0.001).

To analyze the spatial and developmental expression of *GALT2* and *GALT5*, RNA was isolated from different organs and tissues and analyzed by qPCR (Fig 6A). Both *GALT* genes were ubiquitously expressed in *Arabidopsis* in an overlapping, but distinct pattern. *GALT2* was highly expressed in root and stem, whereas *GALT5* was highly expressed in stem, root and leaf. These findings are consistent with data from publicly available expression databases (S5 Fig). Transcriptomics analysis using GeneCAT (http://genecat.mpg.de) [47], Genevestigator (http://www.genevestigator.com/gv/) [48] and the Arabidopsis eFP browser (http://bar.utoronto.ca/efp/cgi-bin/efpWeb.cgi) [49] indicate both genes are widely expressed with *GALT5* having a higher overall expression compared to *GALT2*. It is also noteworthy that these databases indicate that *GALT5* is highly expressed in mature pollen (S5A and S5C Fig).

### Compensatory mechanism of *GALT2* and *GALT5*

To investigate whether transcriptional compensation occurs between *GALT2* and *GALT5* or with the other four members of the Arabidopsis GT-31 family encoding both GALT and GALECTIN domains, qPCR analysis was conducted using the *galt2*, *galt5* and *galt2 galt5* mutants (Fig 6B and 6C). While *GALT2* and *GALT5* transcripts were absent in their respective mutant lines, significant increases in the abundance of *GALT4*, *GALT5*, and *GALT6* transcripts in the *galt2* mutants and *GALT2*, *GALT3*, *GALT4*, and *GALT6* transcripts in the *galt5* mutants were observed. The compensation mechanism was also observed in the *galt2 galt5* double mutants, with increases in the abundance of *GALT6*, *GALT4*, and *GALT3* transcripts over that seen in the single mutants. These results indicate transcriptional compensation within this GT-31 clade with the notable exception of *GALT1*, whose expression is unchanged in the mutant backgrounds. Interestingly, *GALT2* and *GALT5* demonstrated the most pronounced increases in transcriptional compensation.

### Biochemical phenotypes of the mutants: GALT activity, β-Yariv-precipitable AGPs and immunolabeling with AGP specific monoclonal antibodies

To provide *in vivo* evidence that *GALT5* encodes an AGP GALT and examine potential functional redundancy with GALT2, the two allelic *galt5* mutants were analyzed along with the *galt2* mutants and the *galt2 galt5* double mutant with respect to GALT activity and content of β-Yariv-precipitable AGPs (Table 1). GALT activity was reduced by 22% and 28% in the two *galt5* mutants compared to wild type plants. This was similar to the 21% and 14% reductions for the *galt2* mutants. Double mutants, however, demonstrated a 34% reduction in activity. In addition, the *galt5* mutants had 40% and 43% less β-Yariv precipitable AGPs compared to wild type, while the *galt2* mutants had reductions of 32% and 35%. The double mutants, however, contained 56% less β-Yariv precipitable AGPs. The profiles of these β-Yariv precipitable AGPs were also examined by HPLC by extracting AGPs from equal amounts of plant material, and revealed that virtually all these AGPs, as opposed to a single or subset of these AGPs, were affected in the single and double mutants, with the double mutant being more severely affected (S6 Fig). It was observed that the AGP peaks in the mutants eluted later and had less protein than the wild type AGP peaks, corresponding to reduced glycosylation.

**Table 1:** GALT activity and amount of β-Yariv precipitated AGPs in WT, *galt2*, *galt5*, and *galt2 galt5* mutants.

Genotype	GALT activity (pmol/hr/mg)	β-Gal Yariv precipitated AGP (μg/g)
WT	6.70 ± 0.79	13.92 ± 3.75
*galt2-1*	5.30 ± 1.20^a^	9.91 ± 2.80^a^
*galt2-2*	5.80 ± 1.01^a^	9.78 ± 3.50^a^
*galt5-1*	5.25 ± 2.20^a^	7.90 ± 6.10^b^
*galt5-2*	4.80 ± 3.50^b^	8.10 ± 3.20^b^
*galt2 galt5*	4.44 ± 0.44^b^	5.83 ± 0.59^b^

Detergent-solubilized microsomal fractions were used for performing a standard GALT assay using [AO]_7_ as the peptide substrate acceptor and UDP-[^14^C]Gal as the sugar donor, and AGPs were extracted, precipitated by β-Yariv reagent, and quantified from 14-d-old plants. The values are averages of at least two independent experiments from two biological replicates. Student’s *t* tests were performed to determine statistical significance (^a^ P < 0.05, ^b^ P <0.01).

In addition, immunofluorescence staining of AGP epitopes was performed to confirm that reduced levels of glycosylated AGPs were present in the *galt2galt5* mutants compared to wild type. The *galt2galt5* double mutant displayed reduced labeling intensity using four AGP specific monoclonal antibodies, JIM4, JIM8, JIM13 and MAC207, in root hairs, pollen tubes and seeds compared to the stronger signals displayed by the corresponding wild type samples (S7 Fig). It should be noted that Arabidopsis pollen tubes do not react with JIM13, as previously reported by Dardelle et al. [41].

### Pleiotropic growth and development phenotypes of the mutants

**Fig 7:**
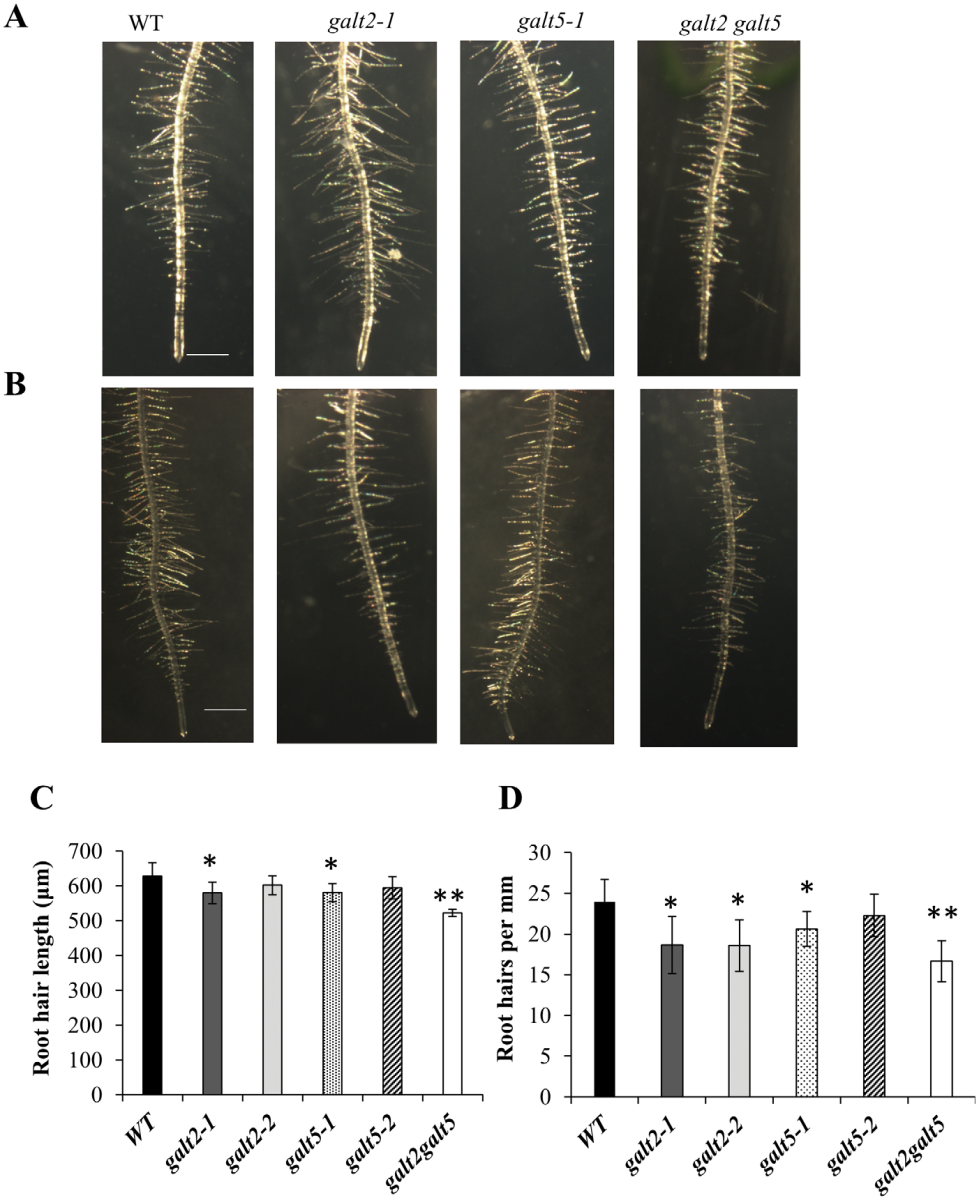
Root hair length and density reduced in the *galt2 galt5* double mutant. (A) Wild type, *galt2-1*, *galt5-1*, and *galt2 galt5* plants were grown on MS agar plates for 10 d with 1% sucrose or (B) with 4.5% sucrose. Bars = 1mm. (C) Quantification of root hair length and (D) density of the *galt* mutants. The asterisks indicate significantly reduced root hair length and density compared with wild type controls according to a Student's t test (*, P < 0.05; **, P < 0.01; n > 300).

**Fig 8:**
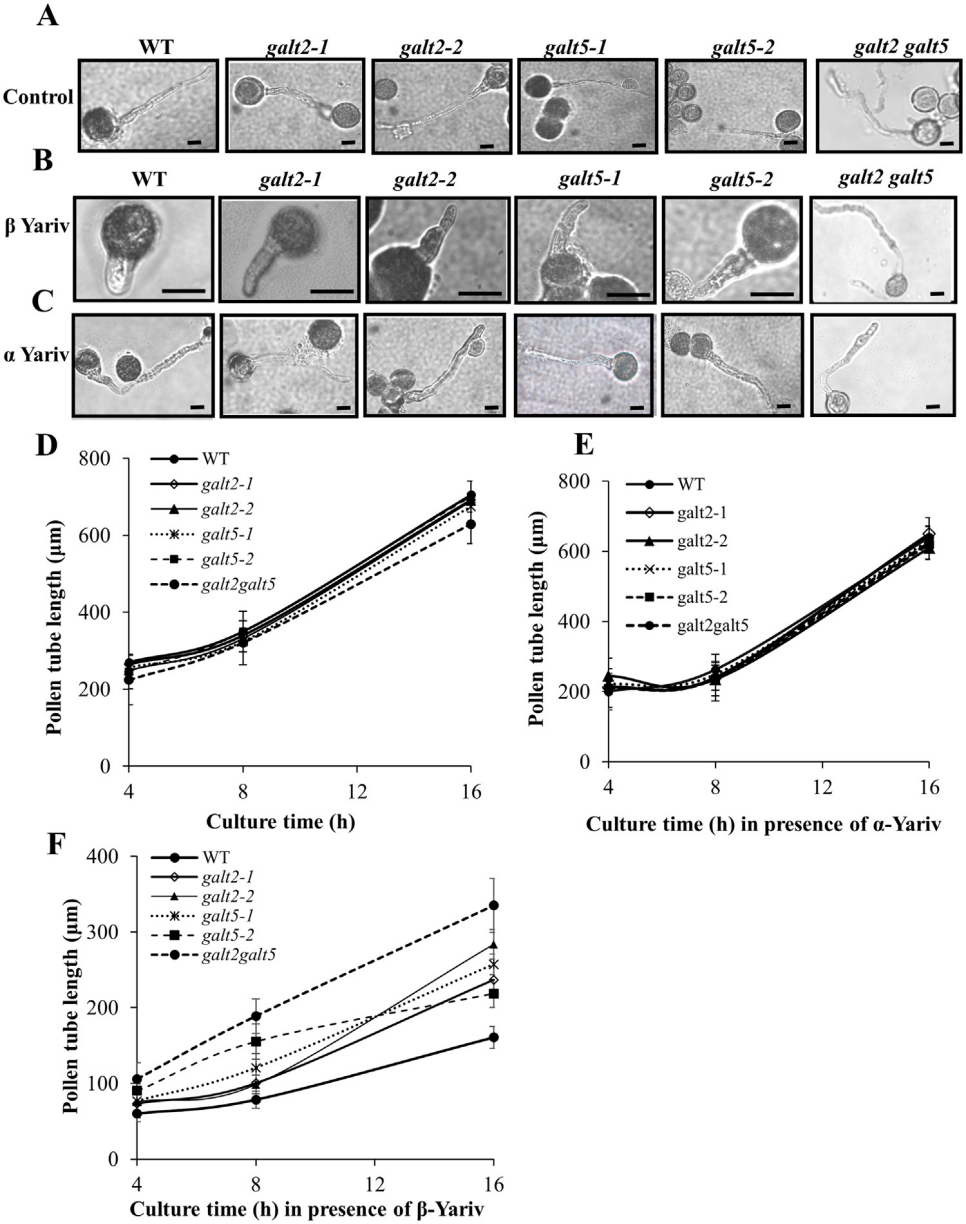
The *galt* single and double mutants demonstrate reduced inhibition of pollen tube growth in response to β-Gal Yariv reagent. (A) Representative images of pollen tubes from wild type, *galt2*, *galt5*, and *galt2 galt5* mutants after 16 h in pollen germination medium, and (B) in pollen germination medium supplemented with 30 μM β-Gal Yariv and (C) in pollen germination medium supplemented with 30 μM α-Gal Yariv reagent. Bar = 30 μm. (D) Pollen tube lengths (from wild type, *galt2*, *galt5*, and *galt2 galt5* plants) were measured over time in the pollen germination medium (E) in pollen germination medium supplemented with 30 μM α-Gal Yariv reagent and (F) in pollen germination medium supplemented with 30 μM β-Gal Yariv reagent. Twenty flowers from each genotype and 25 pollen tubes from each flower were measured using Image J. The experiment was done in triplicate and the values were subjected to statistical analysis by ANOVA, followed by the Tukey's honestly significant difference test. In response to β-Gal Yariv reagent, WT pollen tubes were significantly shorter than pollen tubes from single mutants (P <0.05) and *galt2 galt5* double mutants (P <0.01).

**Fig 9:**
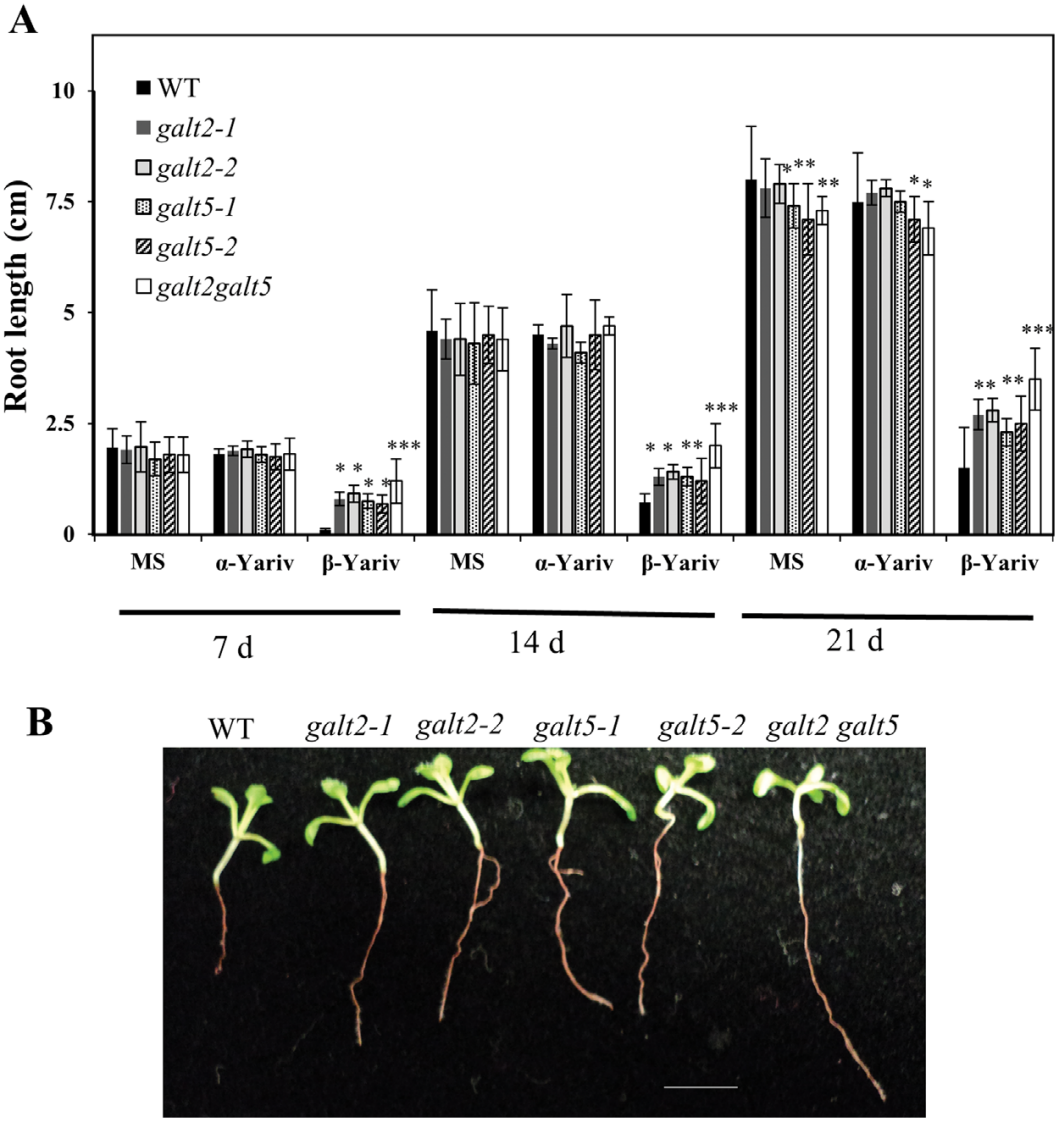
Reduced inhibition of primary root growth of *galt2*, *galt5* and *galt2 galt5* mutants in the presence of β-Gal Yariv reagent. (A) Root lengths of WT, *galt2*, *galt5*, and *galt2 galt5* plants were measured 7, 14 and 21 d after germination and seedling establishment for 5 d on MS plates, on MS plates supplemented with 50 μM α-Gal Yariv reagent, and on MS plates supplemented with 50 μM β-Gal Yariv reagent. Statistical differences were determined by one way ANOVA, followed by the Tukey's honestly significant difference test. Asterisks represent the statistical significance between genotypes (*, P < 0.05; **, P < 0.01; ***, P <0.001) within a treatment group. Vertical bars represent mean ± SE of the experimental means from at least three independent experiments (n = 5), where experimental means were obtained from 10 to 15 seedlings per experiment. (B) Representative images of WT, *galt2*, *galt5*, and *galt2 galt5* plants after 14 d of growth on MS plates supplemented with 50 μM β-Gal Yariv reagent. (C) Representative images of WT, *galt2*, *galt5*, and *galt2 galt5* plants after 14 d of growth on MS plates supplemented with 50 μM α-Gal Yariv reagent. Size bar = 1 cm.

While the *galt2* and *galt5* single mutants were largely indistinguishable from wild type, the double mutants displayed several altered phenotypes related to growth and development under normal growth conditions (Table 2, Figs 7–9, S8 and S9 Fig). Such alterations were reflected in the larger number of rosette leaves, increased flowering time, reduced silique length, and reduced plant height (Table 2, S8 Fig). Root hair length and density were reduced in some single mutants and in the double mutant (Fig 7). Specifically, *galt2-1* and *galt5-1* showed a reduction in root hair length, as did the double mutant, while root hair density was reduced in *galt2-1*, *galt2-2*, and *galt5-1* along with the double mutant. Root growth was also inhibited in some single mutants (*galt5-1* and *galt5-2*) and in the double mutant (Fig 9). In addition, pollen tube growth was slightly inhibited in the double mutant (Fig 8) and frequently associated with disruption of pollen tube tip growth (S9 Fig). Several conditional phenotypes were also examined and revealed marked differences in the mutants as described in the next section.

**Table 2:** Comparisons of various developmental phenotypes displayed by WT, *galt2*, *galt5*, and *galt2 galt5* mutant plants.

Genotype	Rosette leaves (#)	Cauline leaves (#)	Flowering time (days)	Silique length (mm)	Plant height (cm)
WT	9.6 ± 0.36	3.6 ± 0.09	22.4 ± 0.49	13.3 ± 0.25	40.20 ± 0.82
*galt2-1*	10.8 ± 0.41	3.4 ± 0.36	23.4 ± 0.45	12.7 ± 0.15	40.42 ± 0.63
*galt2-2*	11.1 ± 0.59	3.8 ± 0.36	23.4 ± 0.32	12.9 ± 0.55	40.35 ± 0.51
*galt5-1*	10.7 ±0.59	3.4 ± 0.34	22.8 ± 1.44	12.4 ± 0.30	40.25 ± 0.46
*galt5-2*	11.0 ± 0.15	3.9 ± 0.60	23.3 ± 0.25	12.9 ± 0.30	40.16 ± 0.32
*galt2 galt5*	18.9 ± 0.83^a^	4.0 ± 0.05	26.9 ± 0.52^a^	10.5 ± 0.18^a^	36.42 ± 0.68^a^

All measurements are means ±SE of 15 to 20 plants per genotype. Statistically significant differences were determined by performing Student’s t tests (P < 0.05) using Graphpad Quickcalcs (http://www.graphpad.com/quickcalcs/). Plants were grown under long day conditions. Plant height and silique length were measured from 40-d-old plants.

### Mutants demonstrate reduced inhibition of pollen tube growth and root growth in the presence of β-Yariv reagent

β-Yariv reagent is known to inhibit pollen tube growth by disrupting AGPs [50–52]. Here, pollen from *galt2* and *galt5* single mutants as well as the double mutant were germinated and grown in β-Yariv reagent (Fig 8). Wild type pollen was used as a control and showed reduced pollen tube growth in the presence of β-Yariv reagent as expected. In contrast, pollen tube growth was less inhibited in the single mutants, and was even less inhibited in the double mutant. In other words, the mutants showed less sensitivity to β-Yariv-induced pollen tube growth inhibition. α-Yariv reagent, which does not bind to AGPs but is similar in structure to β-Yariv reagent, was used as another control treatment in these experiments and produced results identical to the unsupplemented control treatment. It should be noted that this experiment also revealed other non-conditional mutant phenotypes in the control treatment, namely pollen tube growth was slightly inhibited in the double mutant (Fig 8) and frequently associated with disruption of pollen tube tip growth (S8 Fig), as mentioned in the previous section.

β-Yariv reagent is equally well known to inhibit root growth by disrupting AGPs [53–55]. Here, *galt2* and *galt5* single mutant seedlings as well as double mutant seedlings were grown in the presence of β-Yariv reagent (Fig 9). Wild type seedlings were used as a control and showed reduced root growth in the presence of β-Yariv reagent as expected. In contrast, the single mutants showed a β-Yariv insensitive root growth phenotype, and the double mutant displayed even greater β-Yariv insensitivity with respect to root growth. α-Yariv reagent was used as another control treatment in these experiments and produced results identical to the unsupplemented control treatment. This experiment also revealed another non-conditional mutant phenotype in the control treatments at 21 d, namely root growth was inhibited in some single mutants (*galt5-1* and *galt5-2*) and in the double mutant (Fig 9), as mentioned in the previous section.

### Mutant seed germination and root growth are hypersensitive to NaCl

**Fig 10:**
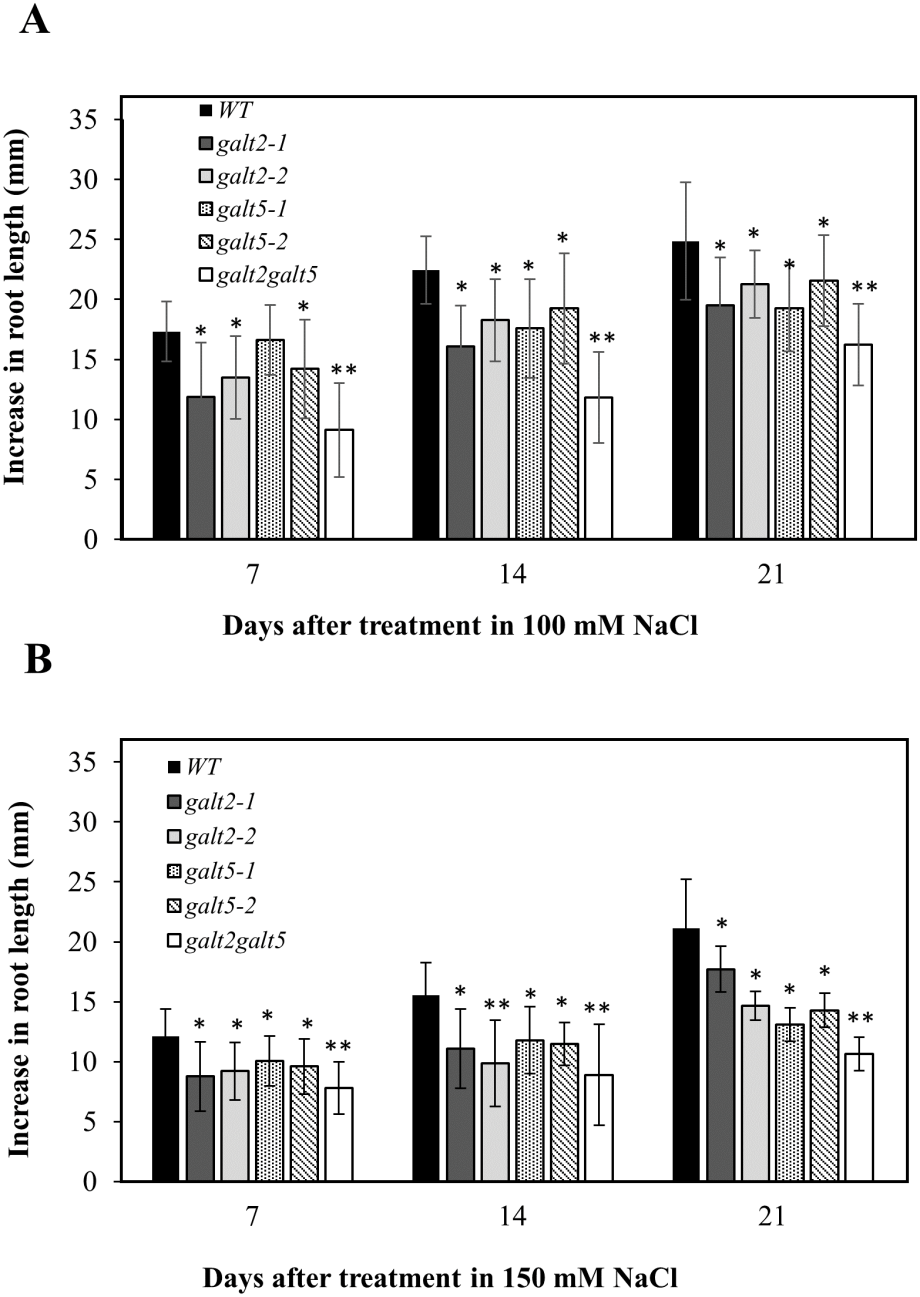
Salt induced inhibition of primary root elongation in *galt2*, *galt5* and *galt2 galt5* mutants. Five-day-old wild-type, *galt2*, *galt5* and *galt2 galt5* seedlings germinated on MS medium were transferred onto media containing (A) 100 mM NaCl or (B) 150 mM NaCl and grown vertically. Root elongation (i.e., increase in length after transfer) was measured after 7, 14 and 21 d of growth. Data are the means ± SE of measurements from five independent experiments (total n = 100). Statistical differences were determined by one way ANOVA, followed by the Tukey's honestly significant difference test (*, P <0.05 and **, P <0.01).

Seed germination and root growth are known to be impaired in response to salt [37]. Here, the double mutants showed a significant reduction in seed germination compared to wild type, while the single mutants showed some reduction in seed germination, which was not statistically significant (S10 Fig). Radicle growth, however, was delayed in the single mutants and even more delayed in the double mutant in response to 100 and 150 mM NaCl (S11 Fig). Root growth was also inhibited in the single mutants, and even more inhibited in the double mutant over a 21 d time course, in response to 100 and 150 mM NaCl (Fig 10 and S12 Fig). This hypersensitive root growth was observed in the presence of varying concentrations of NaCl, KCl, and LiCl, but not in the presence of CsCl and mannitol (S13 Fig).

**Fig 11:**
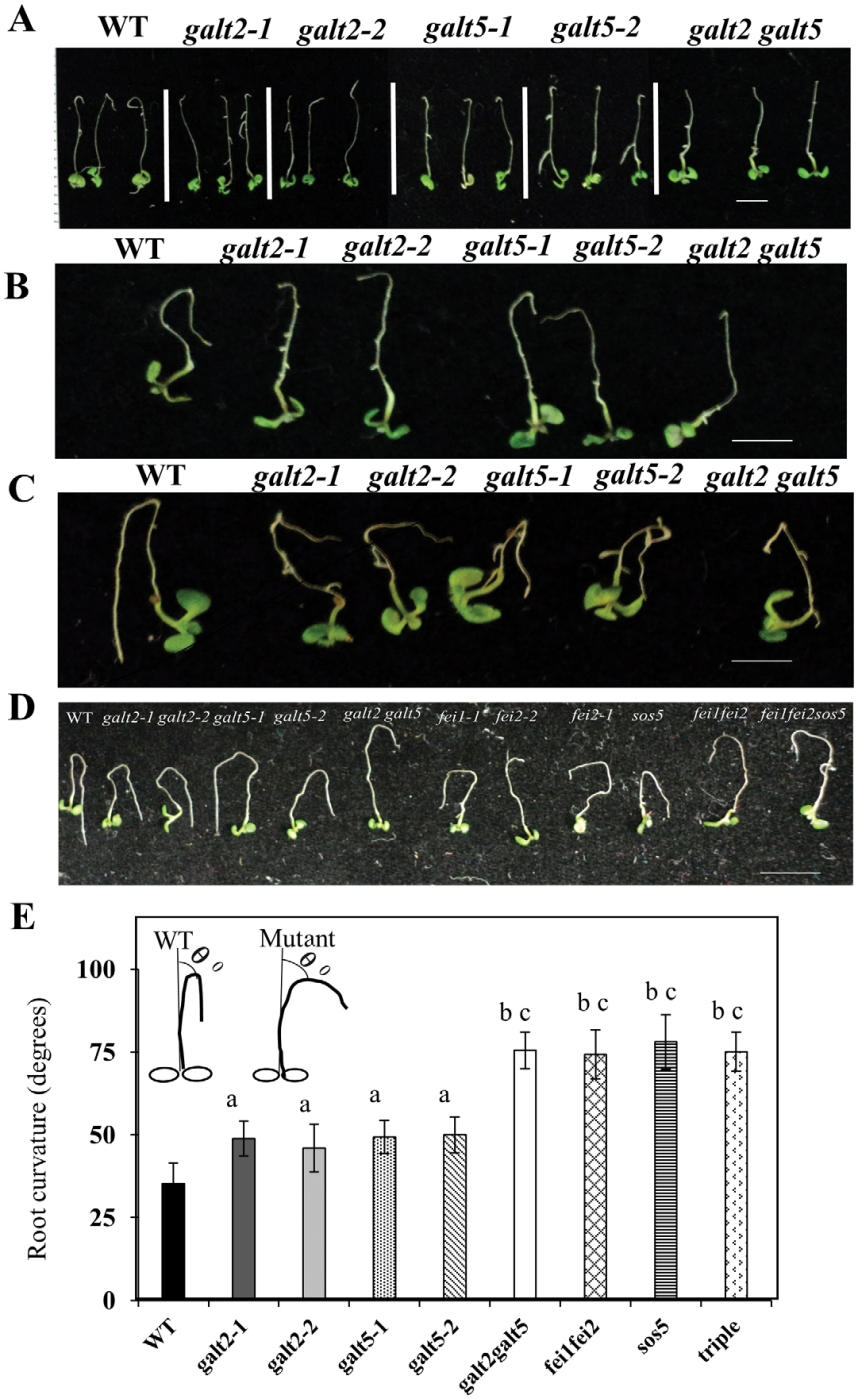
Root-Bending assay of wild type, *galt*, *sos5*, and *fei* mutant seedlings. Five-day-old seedlings grown on MS plates were transferred to MS plates with 100 mM NaCl and reoriented at an angle of 180° (upside down). The photographs were taken 3 d (A), 5 d (B) and 10 d (C and D) after seedling transfer. Bar = 10 mm. (E) Analysis of root curvature in WT, *galt*, *fei1*, *fei2* and *sos5* mutant plants. Statistical differences were determined by one way ANOVA and ‘a’ denotes a significant difference of root curvature (P<0.05) between WT and single *galt* mutants, ‘b’ denotes a significant difference of root curvature (P<0.01) between *galt* single mutants and *galt2 galt5*, *fei1fei2*, *sos5* and *fei1fei2sos5* mutants, and ‘c’ denotes a significant difference of root curvature (P<0.001) between WT and *galt2 galt5*, *fei1fei2*, *sos5* and *fei1fei2sos5* mutants. Vertical bars represent mean ± SE of the experimental means from at least two independent experiments (n = 5), where experimental means were obtained from 15 seedlings per experiment.

Single and double mutants were also subjected to a root-bending assay, which is routinely used to screen salt-hypersensitive mutants or transgenic plants [37]. WT plants readily reoriented root growth, whereas the single mutants showed delayed root bending with the double mutants showing a greater delay (Fig 11). Other known salt-hypersensitive mutants, including *sos5*, which encodes a fasciclin-like AGP called FLA4, and *fei1* and *fei2*, which encode cell wall receptor like kinases which interact with *sos5*, were also tested in this root bending assay as they may be related to *galt2* and *galt5* and showed various degrees of delayed root bending [24], [56] (Fig 11D). The angle of root curvature in the single mutants and to a larger extent in the double mutants were observed to be greater than that of the WT due to their delayed response towards salt stress (Fig 11E).

**Fig 12:**
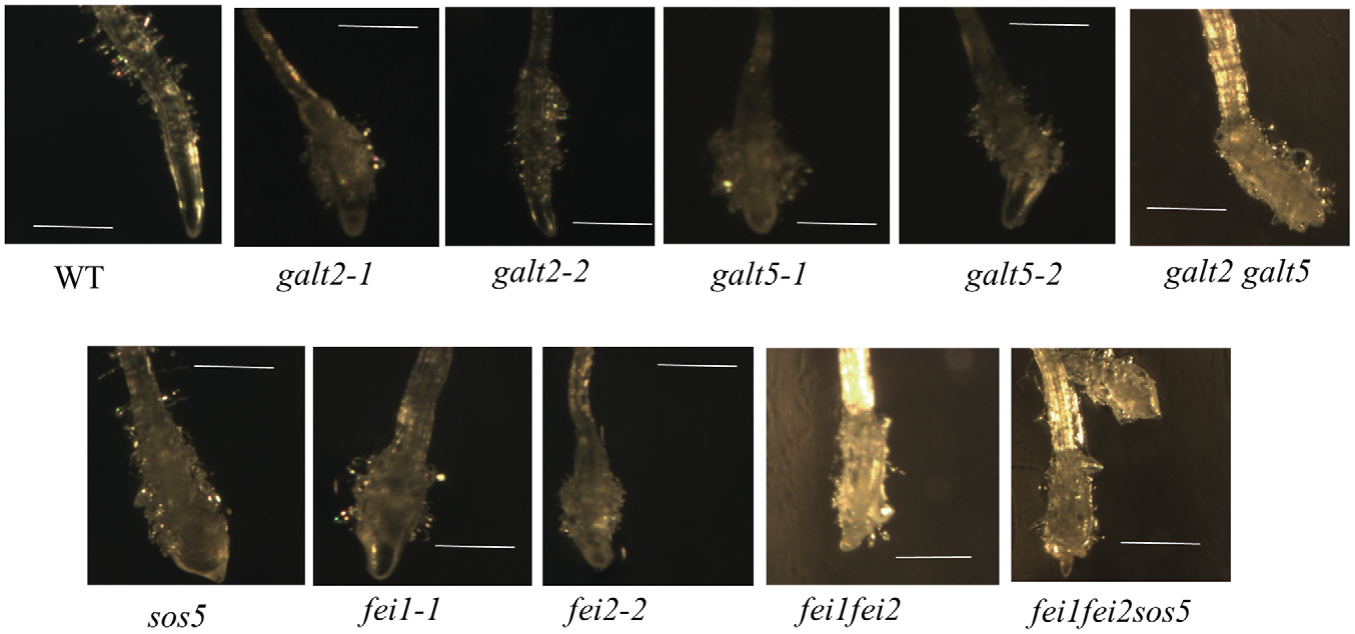
Conditional root anisotropic growth defects of *galt*, *sos5*, and *fei* mutants. Light microscopic images of root tips of plant seedlings from indicated genotypes grown for 10d in MS plates with 100 mM NaCl. Seeds were germinated in MS plates and grown for 3d before transferring to the MS plates with 100 mM NaCl. Bar = 1mm.

Root tip swelling in response to 100 mM NaCl was observed in the *galt* single mutants; this swelling was even more pronounced in the double mutant (Fig 12). Other mutants, including *sos5*, *fei1*, *fei2*, *fei1fei2*, and *fei1fei2sos5*, were also examined and demonstrated root tip swelling in response to salt as reported previously [56].

### Double mutant (*galt2 galt5*) displays less seed coat mucilage

**Fig 13:**
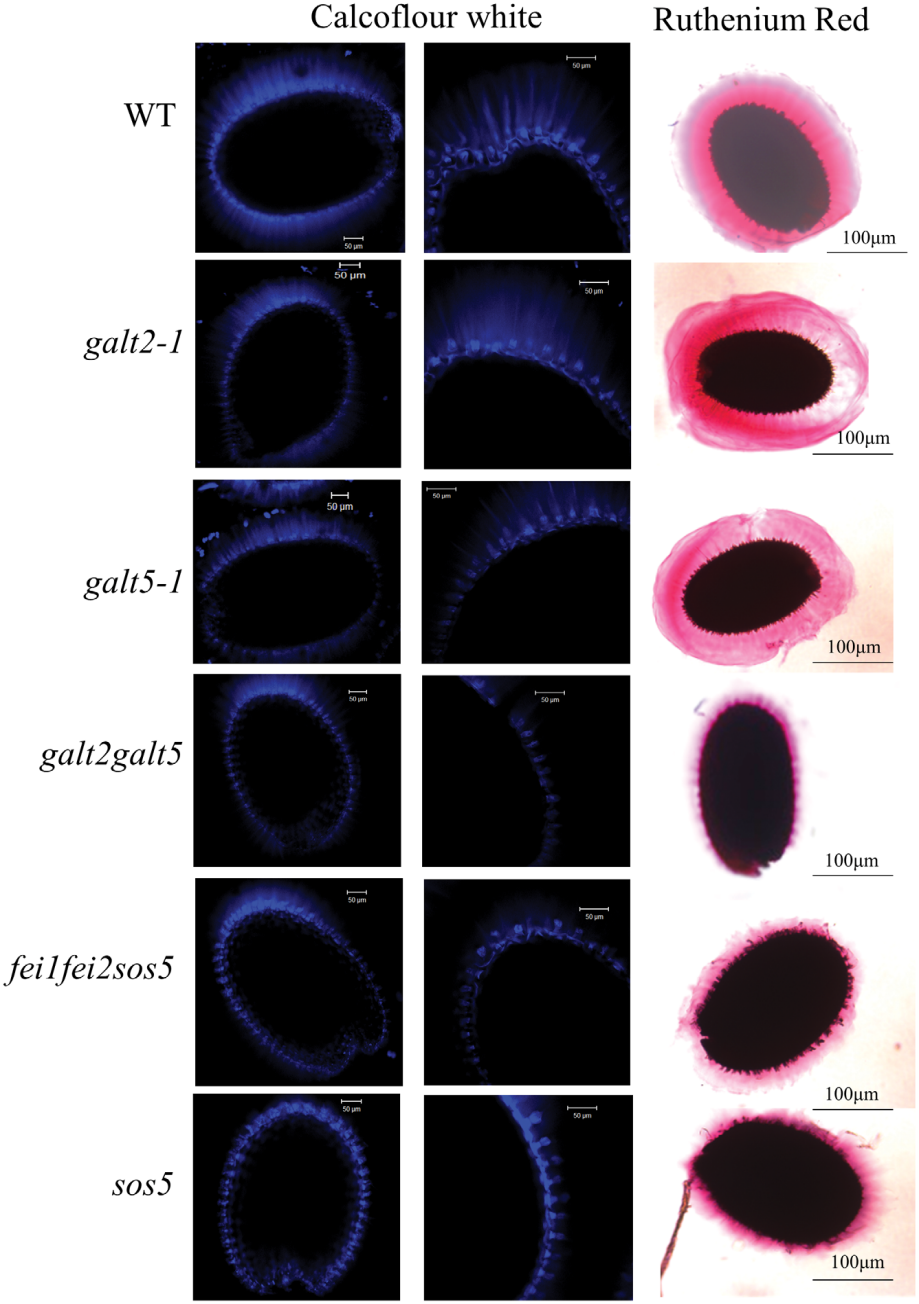
Staining of seed coat mucilage for cellulose and pectin in wild type, *galt*, *sos5*, and *fei* mutant seeds. Seeds of the indicated genotypes were prehydrated with water and stained with Calcofluor white and ruthenium red to visualize cellulose and pectin with a Zeiss LSM 510 META laser scanning confocal microscope.

Calcoflour white, which stains cellulose, and ruthenium red, which stains pectin, were used to stain *galt* single and double mutant seeds along with *sos5* and *fei1fei2sos5* mutant seeds to examine seed coat mucilage (Fig 13). The double mutant displayed reduced cellulose ray staining and reduced pectin staining in the mucilage adhering to the seeds compared to the wild type and *galt2* and *galt5* single mutants. This double mutant seed phenotype was similar to that displayed by *sos5* and *fei1fei2sos5* [26], [47 39].

## Discussion

### GALT5 is an AGP Hyp-GALT and other AGP glycosyltransferases

Biochemical and genetic evidence are presented here indicating that GALT5, similar to GALT2, functions as an AGP-Hyp-*O*-galactosyltransferase [17]. Detergent permealized microsomal preparations from *Pichia* cells expressing *GALT5* exhibit Hyp-GALT activity, catalyzing transfer of [^14^C]Gal from UDP-[^14^C]Gal onto both a chemically synthesized peptide [AO]_7_ and endogenously produced HF-deglycosylated d[AO]_51_ substrate acceptors (Fig 1). Product characterization revealed that a single Gal residue is transferred to Hyp residues, as was the case for GALT2 (Fig 2). This observation is consistent with the hypothesis that *O*-glycosylation in plants occurs by the stepwise addition of sugar residues, as opposed to en block transfer that is characteristic of *N*-glycosylation.

Hyp-GALT activity observed here using heterologously expressed *GALT5* in *Pichia* is considerably lower than observed using plant microsomes, but is consistent with our previous findings with *GALT2* [17], [29]. One possible explanation for this could be that multiple Hyp-GALT enzymes, multi-enzyme complexes, and/or plant-specific cofactors are involved in the biosynthesis of AGP glycans, which are absent in *Pichia* cells.

Substrate specificity of GALT5 was investigated using various potential acceptor substrates and demonstrated that GALT5 is specific for AGP sequences (Fig 3). These findings are consistent with the Hyp contiguity hypothesis, which states that clustered, non-contiguous Hyp residues are sites of arabinogalactan polysaccharide addition, whereas contiguous Hyp residues are sites for the addition of Ara oligosaccharides [57], [58]. Heterologously expressed *GALT5* in *Pichia* microsomes has similar biochemical properties to the GALT(s) present in Arabidopsis microsomal membranes and GALT2 (S4 Fig) [17], [29]. GALT5 specifically requires UDP-Gal as the sugar donor, has a pH optimum of 6.5 (in contrast to 7 for plant microsomes), and has a requirement for Mg^2+^ and Mn^2+^ (in contrast to Mn^2+^ for plant microsomes) for its optimal activity. These differences are likely a reflection of studying the properties of a single GALT enzyme in *Pichia* microsomes in contrast to the more complex GALT enzyme mixture in Arabidopsis microsomes that includes plant-specific factors and is consistent with the biochemical characteristics of GALT2 [17].

Genetic mutant analysis provides additional *in vivo* evidence that GALT5 functions as an AGP-Hyp-GALT, which is functionally redundant to GALT2 (Table 1). Two allelic *galt5* knock-out mutants have reduced Hyp-GALT activity and contain considerably less glycosylated (i.e., β-Yariv precipitiable) AGPs. Allelic *galt2* knock-out mutants demonstrate similar biochemical phenotypes, while *galt2 galt5* double mutants possess even less enzyme activity and glycosylated AGPs compared to the single mutants. In addition, HPLC AGP profiling of the *galt2*, *galt5*, *and galt2 galt5* mutants extends these finding and indicates that GALT2 and GALT5 activity is not limited to a particular AGP or a small subset of AGPs, but instead broadly act on coexpressed AGPs (S6 Fig). Furthermore, immunofluorescent labeling of AGPs in root hairs, pollen tubes and seeds was used to demonstrate that elimination of GALT2 and GALT5 led to the biosynthesis of AGPs with reduced glycosylation in different organs (S7 Fig). This finding is consistent with the reduced GALT activity and amounts of β-Yariv precipitiable AGPs in the *gat2galt5* mutants as well as the HPLC profiling of the AGPs obtained from the *galt2galt5* mutants. In this context, it should be noted that *GALT2* and *GALT5* have overlapping patterns of gene expression and demonstrate transcriptional compensation when either one or both genes are knocked out (Fig 6 and S5 Fig).

**Fig 14:**
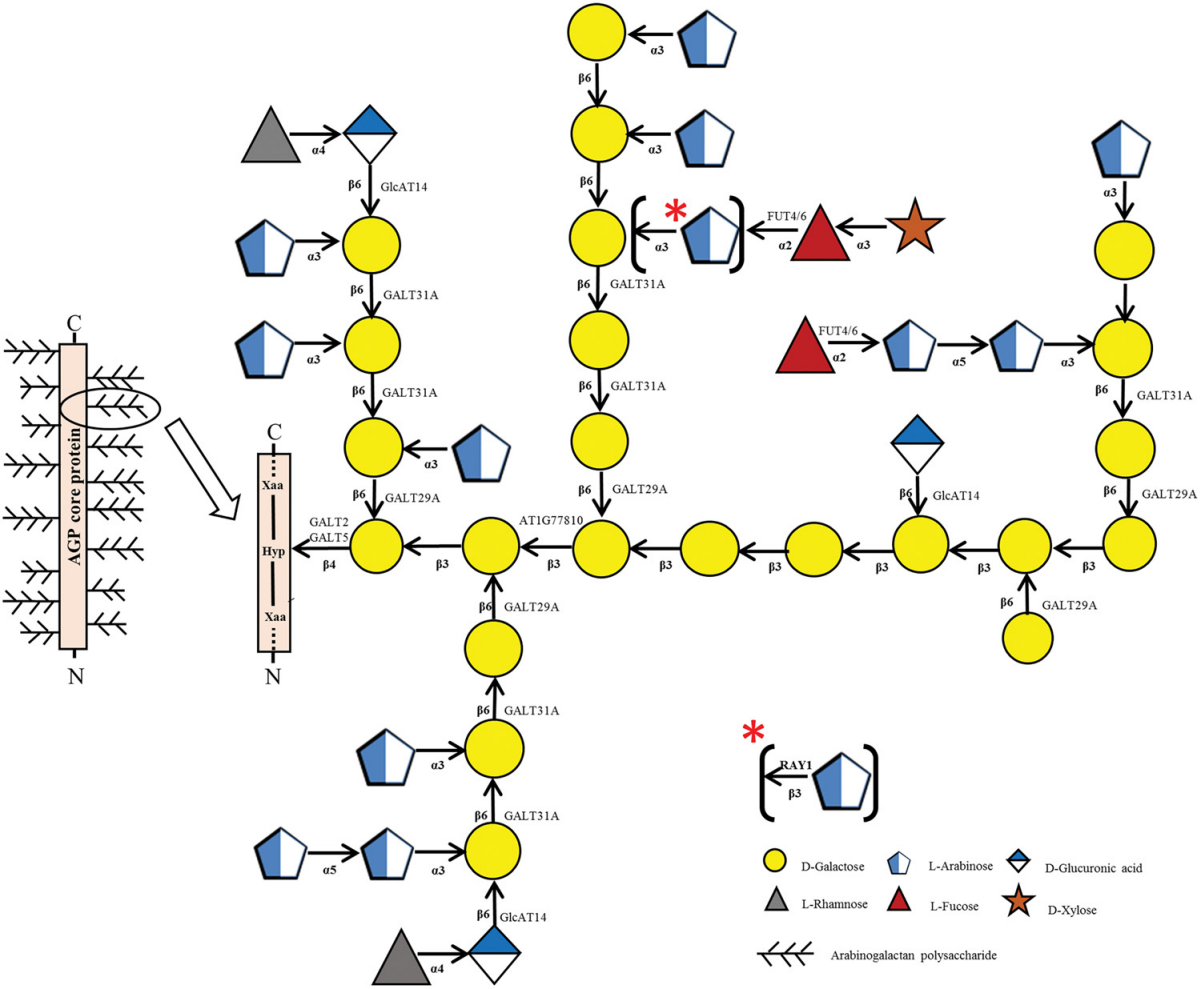
Sites of action of known glycosyltransferases acting on AGPs are depicted within a representative AGP glycomodule sequence found within an AGP molecule. This glycomodule structure is based on information presented by Tryfona et al. [81]. Additional details on each of the known glycosyltransferases are listed in S1 Table.

Identification of GALT5 as an AGP Hyp-GALT adds to the growing list of the enzymes responsible for AGP glycosylation (Fig 14 and S1 Table). Currently, there is biochemical and/or genetic evidence for eleven AGP glycosyltransferases residing in multiple GT families, including two Hyp-*O*-GALTs in GT31 (GALT2 and GALT5), one β-1,3-GALT in GT31 (At1g77810), one β-1,6-GALT in GT31 (GALT31A), one β-1,6-GALT in GT29 (GALT29A), three β-1,6-GlcATs in GT14 (GlcAT14A, GlcAT14B, GlcAT14C), two α-1,2-FUTs in GT37 (FUT4 and FUT6), and one β-AraT in GT77 (RAY1). Several AGP glycosyltransferases, however, remain to be cloned and identified, including α-AraTs, α-rhamonsyltransferases, and α-xylosyltranferases, as well as additional enzymes related to those listed above. It will be particularly interesting to know the number of enzymes responsible for synthesizing the β-1,3 and β-1,6 galactose chains and their detailed substrate specificities, particularly with respect to processivity. It is unknown whether these enzymes exist in a large biosynthetic complex, although a recent study reports that GALT31A and GALT29A interact with one another [20].

### GALT5 is localized to Golgi vesicles

GALT5 was localized to Golgi vesicles, consistent with bioinformatics predictions using Signal P and Golgi predictor, biochemical pulse-chase experiments of HRGP biosynthesis [59], [60] a proteomics technique for localization of organelle proteins by isotope tagging [60], and localization studies performed with other AGP GTs, including GALT2, AT1G77810, GALT31A, GALT29A, GlcAT14A, and FUT6 (Fig 4 and S2 Table). Interestingly, GALT2 as well as Hyp-GALT activity was identified in the ER as well as the Golgi, indicating AGPs likely initiate Hyp galactosylation in the ER and continue to be Hyp galactosylated and further glycosylated in the Golgi [17], [29], [61 62]. A recent study has also localized GALT31A, GALT29A, and GlcAT14A to unique subcellular compartments, which are not part of the trans-Golgi network, cis-Golgi network or endosomes [63].

### AGP glycosylation required for normal growth and development: GALT and AGP glycosyltransferase mutant phenotypes

While single *galt2* or *galt5* mutants are largely indistinguishable from wild type with respect to their non-biochemical phenotypes, *galt2 galt5* double mutants are clearly compromised with respect to normal growth and development (Table 2, Figs 7–9, S8 and S9 Fig). Given that the single mutants have biochemical phenotypes corresponding to a reduction in AGP glycosylation that is exacerbated in the double mutant, it is reasonable to conclude that critical threshold levels of glycosylated AGPs are required for normal growth and development. In other words, single mutants have sufficient levels of glycosylated AGPs to appear normal, while double mutants do not and display pleiotropic phenotypes affecting the growth and development of roots, leaves, inflorescences, flowers, pollen, and seeds. Specifically, *galt2 galt5* double mutants display shorter roots (Fig 9), shorter and less dense root hairs (Fig 7), more rosette leaves (Table 2), shorter inflorescences (Table 2), delayed flowering (Table 2), shorter pollen tubes (Fig 8), disruption of pollen tips (S9 Fig), and reduced seed coat mucilage (Fig 13). These observations also lend further support to the notion that GALT5 and GALT2 are functionally redundant. More severe growth and development consequences, including lethality, are likely as additional Hyp-GALT genes are identified and cumulatively knocked-out.

In contrast, other mutant phenotypes were observed for some of the known AGP glycosyltransferases (S1 Table). Specifically, mutants for the β-1,6-GALT (GT31A) were embryo lethal [19], mutants for one of the β-1,6-GlcATs (GT14A) displayed longer roots [21], and mutants for the β-AraT in GT77 (RAY1) had longer hypocotyls [23]. Without knowing the precise site(s) of enzyme action and the biochemical extent to which each of these mutants alters AGP structure, it is difficult to interpret the functional implications of these mutant data. One possibility is that specific glycomodules within the AG polysaccharide are responsible for specific functions.

Roots and root hairs are particularly sensitive to the loss of glycosylated AGPs since some of the single *galt* mutants display more subtle versions of the phenotypes observed in *galt2 galt5* double mutants. Such root hair sensitivity was previously observed in mutants for proline hydroxylation, extensins, and extensin arabinosylation, which display impaired growth [64], [65].

### AGP glycosylation required for root and tip growth

Several conditional phenotypes also characterize the *galt* single and double mutants; the most interesting of which involve alterations in root growth, pollen tubes, and root hairs in response to β-Yariv or NaCl treatments. In roots, β-Yariv binds AGPs, specifically to their β-1,3-galactan chains [11], and inhibit root elongation (Fig 9) [53–55]. This inhibition is alleviated in the single and double mutants, which have reduced AGP glycosylation. This conditional phenotype indicates a role for AG polysaccharides in root elongation; this role is largely, but not completely masked in the mutants under normal growth conditions due to gene redundancy. In particular, normal root growth is inhibited in the double mutant, which corroborates a function for AGP glycosylation in root elongation. Pollen tube growth is affected in a similar manner. In pollen, β-Yariv binds to AGPs and inhibits pollen tube growth (Fig 8) [49–51]. This inhibition is alleviated in single and double mutants, which likely have reduced AGP glycosylation. This conditional phenotype indicates a role for AG polysaccharides in pollen tube growth; as in the roots, this role is largely, but not completely masked in the mutants under normal growth conditions due to gene redundancy. Notably, under normal growth conditions, the double mutants show reduced pollen tube growth, as well as reduced root hair growth. These results are consistent with observations that knock-out mutants of pollen-specific AGP genes (*AGP6*, *AGP11* and *AGP40*) lead to impaired pollen tube elongation [66] and that knock-out mutants of prolyl 4-hydroxylase genes (*P4H2*, *P4H5*, *P4H13*) display shorter root hairs [64]. Taken together, these studies indicate the important functional contribution of the carbohydrate moiety of AGPs to root and pollen tube growth and development, particularly with respect to polarized tip growth.

Salt treatment was also used to reveal conditional phenotypes in the single and double mutants. In roots, NaCl treatment results in reduced root growth [67–69], and the root bending assay can be used to screen for salt sensitivity [37]. Here, the single and particularly the double mutants are salt hypersensitive, demonstrating significantly reduced root growth and delayed root bending, further corroborating the functional contribution of the carbohydrate moiety of AGPs to root growth (Figs 10 and 11). Moreover, root elongation in the single and double *galt* mutants was hypersensitive to NaCl, LiCl, and KCl, but not to CsCl, as was previously observed for the *sos5* mutant (S13 Fig) [24]. Single and double mutants for *FUT4* and *FUT6* also demonstrate reduced root growth in response to salt treatment, illustrating more specifically the importance of fucose in the AG side chains in root growth [70], [71]. In addition, NaCl treatment can result in root tip swelling in salt hypersensitive mutants. The single and double *galt* mutants also display this phenotype. Interestingly, mutants for SOS5, a fasciclin-like AGP, as well as two cell wall receptor like kinases (FEI1 and FEI2) which are in the same genetic pathway as SOS5, phenocopy the *galt2*, *galt5*, and *galt2 galt5* mutants with respect to NaCl—induced root tip swelling and salt hypersensitivity in the root bending assay.

### GALTs and cellular signaling

AGPs are implicated as cellular signaling molecules [24], [72], [73]. Such implications stem from the observation that many AGPs have the capacity to be GPI-anchored to the plasma membrane and contain information-rich AG polysaccharides which could serve as signals themselves or bind to signaling molecules, such as calcium, and/or signal transduction molecules, such as plasma membrane/cell wall kinases. One of the more compelling indications that AGPs function in cellular signaling centers on SOS5/FLA4, a GPI-anchored fasciclin-like AGP; *sos5* mutants are known to be salt hypersensitive and exhibit root tip swelling [24]. This root tip swelling phenotype is phenocopied by *fei1fei2* mutants, which lack a pair of cell wall receptor-like kinases, FEI1/FEI2, and are defective in cellulose biosynthesis [54], [74]. Moreover, genetic evidence indicates SOS5 and FEI1/FEI2 act in the same pathway [54]. More recent work in seeds, has indicated SOS5 and FEI2 act in a pathway to synthesize seed coat mucilage, including pectin and cellulose, but the mechanistic details remain to be elucidated [26], [39]. The observations here that the *galt2 galt5* double mutant phenocopies the root swelling phenotypes (Fig 12) as well as the seed coat mucilage and cellulose-deficient phenotypes (Fig 13) of *sos5*, *fei1fei2*, and *sos5fei1fei2* likely indicates that the carbohydrate moiety of SOS5/FLA4 is important for cellular signaling. Experiments are in progress in our lab and elsewhere to determine whether the AG polysaccharides of SOS5 are responsible for directly or indirectly interacting with the extracellular domains of FEI1 and FEI2 and thereby activating the intracellular kinase domains. Such interactions may be similar to that already discovered between pectin and wall-associated kinases [75].

### Concluding remarks

AGPs are long known to be expressed throughout the plant kingdom and implicated to function in various aspects of plant growth and development. This work identifies two key enzymes, GALT5 and GALT2, responsible for *O-*glycosylation of AGPs and elucidates the importance of the carbohydrate moiety in carrying out such functions. The detailed mode of action by which the AG polysaccharides trigger such growth and developmental events, however, remains to be determined. It is unknown whether the multitude of defects can be traced to a common mode of action or multiple modes of actions. For example, alterations to the AG polysaccharides could prevent intermolecular interactions within the wall and lead to a loss of cell wall integrity. This brings to mind the recent finding that AGPs can serve a structural role in crosslinking pectin and hemicellulose [76] as well as earlier suggestions that AGPs assist with the packaging and delivery of cell wall cargo in Golgi-derived secretory vesicles [77] or act as water laden, plasma membrane-cell wall shock absorbers [7]. Alternatively, such AG polysaccharide alterations may disrupt cellular signaling by preventing interactions with signaling molecules or signal transduction molecules. For example, AG polysaccharides can act in cellular signaling by their ability to bind calcium, to serve as cell wall integrity sensors, and/or to act as co-receptors which interact with signaling molecules such as kinases, particularly those located at the cell surface [9], [10], [78–80]. Future research designed to elucidate the molecular interactions between the AG polysaccharides and other molecules at the cell surface promises to hold the key to understanding the mode of action for these intriguing molecules.

## Supporting Information

S1 Fig Domain organization and sequence alignment of the GALT5 and GALT2 proteins. (A) Schematic diagram of GALT5 and GALT2 with their conserved domains as determined by Prosite (http://prosite.expasy.org/); the image was prepared using Prosite MyDomains (http://prosite.expasy.org/cgi-bin/prosite/mydomains/). The N-terminal transmembrane, GALECTIN (Pfam PF00337) and GALT (Pfam PF01762) domains are denoted by green, yellow and red structures. The solid blue triangles denote DXD motifs. (B) Protein sequence alignment of GALT5 and GALT2. Sequences were aligned using the PRALINE multiple sequence alignment program at http://www.ibi.vu.nl as described by Pirovano et al. [1]. Both the proteins have a single membrane spanning domain (GALT5, residues 27–49 and GALT2, residues 70–92), a GALECTIN domain (GALT5, residues 194–392 and GALT2, residues 248–461), and a GALT domain (GALT5, residues 465–672 and GALT2, residues 508–693). GALT5 and GALT2 showed 57% identity and 79% similarity in their protein sequences.
(EPS)

S2 Fig Screening for the presence of 6x His-tagged GALT5 and GALT activity in transformed *Pichia* cell lines. (A) Western blot analysis of the 6x His-tagged GALT5 protein in *Pichia* microsomal membrane preparations using the His antibody. C1 to C5 designate five independent cloned lines of transgenic *Pichia* cells transformed with the *6x His-GALT5* gene construct. A *Pichia* cell line transformed with the empty expression vector was used as the negative control (NC). (B) [AO]_7_-dependent GALT activity tests of the five transgenic *Pichia* cell lines using Triton X-100 permeablized microsomal membranes. For each line, 250 μg of total microsomal membrane protein was used for the assay. [^14^C]Gal radiolabel incorporation is expressed as pmol/h/mg protein and reflects the difference between total incorporation obtained in reaction products in the presence versus absence of [AO]_7_ acceptor substrate. Reactions were done in triplicate and mean values ±SE are presented. All cell lines tested had GALT5 activity but varied in the rate of incorporation. Student’s *t-*test was performed using Graphpad Quickcalc (http://www.graphpad.com/quickcalcs/) and significant differences in GALT activity were detected with respect to NC (*, P <0.05 and **, P <0.01).
(EPS)

S3 Fig Monosaccharide analysis of the RP-HPLC purified [AO]_7_:GALT5 reaction product following acid hydrolysis. Permeablized microsomal membranes from the transgenic *Pichia* C5 line expressing 6x His-GALT5 served as the enzyme source in the [AO]_7_:GALT reaction. The [^14^C]-labeled monosaccharides were analyzed by High-Performance Anion-Exchange Chromatography (HPAEC) on a CarboPac PA-20 column. Elution times of monosaccharide standards are as indicated with arrows at the top.
(EPS)

S4 Fig Biochemical characterization of the [AO]_7_:GALT5 activity. Data presented are an average of duplicate assays. A. Specificity of the GALT5 enzyme for nucleotide sugar donors was analyzed by monitoring incorporation of [^14^C]radiolabeled galactose onto [AO]_7_ substrate acceptor in presence of UDP-[^14^C]Glc, UDP-[^14^C]Gal, UDP-[^14^C]Xyl, and GDP-[^14^C]Fuc. (B) Effect of pH on enzyme activity. (C) Effect of different divalent ions (5 mM) on enzyme activity.
(EPS)

S5 Fig Expression profiles of GALT2 and GALT5 in publicly available databases. Expression profiles of *GALT2* and *GALT5* as depicted by (A) GeneCAT, (B) Genevestigator and (C) eFP browser. Both genes display expression in root and mature pollen as denoted by red arrows.
(EPS)

S6 Fig Profiles of AGPs extracted from WT, *galt2*, *galt5* and *galt2 galt5* mutants and separated by RP-HPLC. HPLC chromatograms of (A) [AO]_7_ peptide (B) AGPs from WT, (C) AGPs from *galt2-1*, (D) AGPs from *galt5-1*,and (E) AGPs from *galt2 galt5*. Arrows indicate the most prominent AGP peaks in the chromatographs.
(EPS)

S7 Fig Immunofluorescent labeling of *galt2galt5* and wild type root hairs, pollen tubes and seeds using AGP specific monoclonal antibodies JIM4, JIM8, JIM13 and MAC207. (A) Confocal microscopy images of WT and *galt2galt5* root hairs (B) pollen tubes and (C) seeds. Significant reduction in signal intensity was observed in the *galt2galt5* samples compared to the WT. All immune-histochemical experiments were repeated twice with ten seedlings for root hairs, ten flowers with five pollen tubes and 50 seeds for each genotype respectively.
(EPS)

S8 Fig Morphological phenotypes of WT, *galt2*, *galt5* and *galt2 galt5* mutants. (A) Phenotypes of the indicated seedlings grown *in vitro* on MS media (B) and adult plants grown in soil during long days. Key developmental stages were monitored according to Boyes et al. [2]. Data presented are combined from three experimental replicates. Bars represent averages of 25 or more plants ± SE (Student's t test, *, P <0.05 and **, P <0.01). **(C)** Plants at 28 d after germination.
(EPS)

S9 Fig Disruption of tip growth in pollen tubes of *galt2*, *galt5* and *galt2 galt5* mutants. Disruption of tip growth in pollen tubes as observed after 8 h of *in vitro* germination of pollen in the germination media. WT pollen tubes did not display tube disruption and were used as a control. Bar = 50 μm.
(EPS)

S10 Fig Effect of different concentrations of NaCl on seed germination efficiency of the mutants. Germination frequencies of WT, *galt2*, *galt5* and *galt2 galt5* seeds on various concentrations of NaCl. Seeds were stratified at 4°C in the dark for 48 h and then transferred at 22°C under continuous white light and were quantified after 5 d. Data are the mean ± SE of three biological replicates according to a Student's t test (*, P < 0.05; **, P < 0.01).
(EPS)

S11 Fig Delayed germination of galt2, *galt5* and *galt2 galt5* seeds in the presence of NaCl by using radicle length as an indication of delayed germination. WT, *galt2*, *galt5* and *galt2 galt5* seeds were germinated on MS medium supplemented with either 100 mM NaCl (A) or 150 mM NaCl (B). Radicle lengths were measured over time and standard errors were calculated from at least three independent experiments, each of which contained 50 seeds of WT and 50 seeds of each mutant. Emergence of the radicle was considered the indication of germination. Radicle length was measured by Motic Image version 3.2.
(EPS)

S12 Fig Phenotypes of *galt2*, *galt5* and *galt2 galt5* seedlings depicting salt hypersensitivity. Five-day-old seedlings of WT, *galt2-1*, *galt2-2*, *galt5-1*, *galt5-2* and *galt2 galt5* were grown on MS medium with 1% sucrose for 3 d. Seedlings were then transferred to MS plates supplemented with 100 mM NaCl, and photographed after 7 d (A), 14 d (B) and 21 d (C). Bar = 1cm.
(EPS)

S13 Fig Sensitivity of *galt2* and *galt5* seedlings to various salt and osmotic stresses as measured by root growth. WT, *galt2-1*, *galt2-2*, *galt5-1*, *and galt5-2* seeds were grown on MS medium with 1% sucrose for 5 d. Root growth was measured 7 d after transferring the seedlings to MS plates supplemented with various concentrations of (A) NaCl, (B) KCl, (C) LiCl, (D) CsCl, and (E) mannitol. Error bars indicate SEs (n = 25). The experiments were repeated at least three times with similar results.
(EPS)

S1 File References for supporting information. (DOCX)

S1 Table Information on the known enzymes, genes, and mutants for AGP glycosylation. (nr, not reported).
(DOCX)

S2 Table List of primers used in this study. (DOCX)
