# Supplementary material for: Two Hydroxyproline Galactosyltransferases, GALT5 and GALT2, Function in Arabinogalactan-Protein Glycosylation, Growth and Development in Arabidopsis
Source: PLoS One. 2015 May 14;10(5):e0125624. doi: 10.1371/journal.pone.0125624 (PMC4431829; doi:10.1371/journal.pone.0125624)
Supplement: S1 File — (DOCX) [file pone.0125624.s014.docx]

**References for supporting information**

1. Pirovano W, Feenstra KA, Heringa J (2008) PRALINE™: a strategy for improved multiple alignment of transmembrane proteins. Bioinformatics 24: 492-497.

2. Boyes DC, Zayed AM, Ascenzi R, McCaskill AJ, Hoffman NE, Davis K, Gorlach J (2001) Growth stage–based phenotypic analysis of Arabidopsis: A model for high throughput functional genomics in plants. Plant Cell 13: 1499-1510.
