# Supplementary material for: Two Hydroxyproline Galactosyltransferases, GALT5 and GALT2, Function in Arabinogalactan-Protein Glycosylation, Growth and Development in Arabidopsis
Source: PLoS One. 2015 May 14;10(5):e0125624. doi: 10.1371/journal.pone.0125624 (PMC4431829; doi:10.1371/journal.pone.0125624)
Supplement: S1 Table — (nr, not reported). (DOCX) [file pone.0125624.s015.docx]

| **Table S1.** Information on the known enzymes, genes, and mutants for AGP glycosylation. (nr, not reported) | | | | | | | |
| --- | --- | --- | --- | --- | --- | --- | --- |
| Gene name | Enzyme | GT Family | Gene  Identifier | Mutants | Localization | Mutant phenotypes | References |
| *GALT2*  *GALT5* | hydroxyproline-*O*-β-galactosyltransferase | GT31 | At4g21060  At1g74800 | *galt2-1* (SALK_117233)  *galt2-2* (SALK_141126)  *galt5-1* (SALK_105404)  *galt5-2* (SALK_115741) | Golgi and ER  Golgi | Reduced root growth under salt stress, radial swelling of root tips, reduced seed mucilage adherence | [17] |
| *AT1G77810* | β-1,3-galactosyltransferase | GT31 | At1g77810 | nr | Golgi | - | [18] |
| *GALT31A* | β-1,6-galactosyltransferase | GT31 | At1g32930 | *galt31A* (FLAG_379B06) | Golgi and also  unique subcellular compartments | Embryo lethal mutant | [19] |
| *GALT29A* | β-1,6-galactosyltransferase | GT29 | At1g08280 | nr | Unique subcellular compartments | - | [20] |
| *GlcAT14A*  *GlcAT14B*  *GlcAT14C* | β-1,6-glucuronosyltransferase | GT14 | At5g39990  At5g15050  At2g37585 | *glcat14a-1*  (SALK_06433)  *glcat14a-2* (SALK_043905) | Golgi and also unique subcellular compartments | Enhanced cell elongation in seedlings | [21], [22] |
| *FUT4* | α-1,2-fucosyltransferase | GT37 | At2g15390 | *fut4-1* (SAIL_284_B)  *fut4-2* (SALK_12530) | nr | Reduced root growth under salt stress | [16], [60], [61] |
| *FUT6* | α-1,2-fucosyltransferase | GT37 | At1g14080 | *fut6-1* (SALK_0783)  and *fut6-2* (SALK_09950) | Golgi | Reduced root growth under salt stress | [16], [60], [61] |
| *RAY1* | β-arabinofuranosyltransferase | GT77 | At1g70630 | *ray1-1* (SALK_053158) *ray1-2* (GABI_001C09) | nr | Reduced root growth and reduced rosette size and inflorescence | [23] |
