# Supplementary material for: Two Hydroxyproline Galactosyltransferases, GALT5 and GALT2, Function in Arabinogalactan-Protein Glycosylation, Growth and Development in Arabidopsis
Source: PLoS One. 2015 May 14;10(5):e0125624. doi: 10.1371/journal.pone.0125624 (PMC4431829; doi:10.1371/journal.pone.0125624)
Supplement: S2 Table — (DOCX) [file pone.0125624.s016.docx]

| **Table S2.** List of primers used in this study | | |
| --- | --- | --- |
| Purpose | Forward | Reverse |
| Cloning |  |  |
| *GALT5* | CGCCGCGGATGCATCATCATCATCATCACATGAAAAAACCCAAATTGTCG | GAGTGTTGTAACATGAGATGATCTAGA |
| Subcellular localization |  |  |
|  | CACCATGAAAAAACCCAAATTGTCGAA | TCTCATGTTACAACACTCAGGCTTG |
| Screening for T-DNA |  |  |
| *galt5-1* | galt5-1RP-TTTCCACTTTCGACAATTTGG | GALT5-1LP- CTAATTACATGGTTTTGCGGG |
| *galt5-2* | GALT5-2RP- TGGGGACATTGTACTTGTTCC | GALT5-2LP- TGGTACGCTTGCAAAATTTTC |
| LBa1.3 | ATTTTGCCGATTTCGGAAC |  |
| RT-PCR |  |  |
| *GALT2* | RTF-  tctttgttgcacttaatccaagaag | RTR-  cataagcttaggctattcaagatgg |
| *GALT5* | RTF-  TATGTGAACACGGAGCTCTTGCATTC | RTR-ACATAAATTACGGCTGTTCAAGATGGA |
| *UBQ 10* | GTCGACCCTTCACTTGGTGT | ATCCTCAAGCTGCTTTCCAG |
| *GALT2* | QPCRF-  tcttagacatcgtcctcttaga | QPCRR-  acacagctggaaattttgcc |
| *GALT5* | QPCRF-ACATAAATTACGGCTGTTCAAGATGGA | QPCRR-CTTATGGGATAAGCTCTTAA |

The grey shaded area denotes the 6x His-tag, the underlined nucleotides in the forward primer column denotes a SacII restriction site, whereas the underlined nucleotides in the reverse primer column denotes an XbaI restriction site for GALT5.
